# Postglacial range expansion of high‐elevation plants is restricted by dispersal ability and habitat specialization

**DOI:** 10.1111/jbi.14390

**Published:** 2022-05-19

**Authors:** Pau Carnicero, Johannes Wessely, Dietmar Moser, Xavier Font, Stefan Dullinger, Peter Schönswetter

**Affiliations:** ^1^ Department of Botany University of Innsbruck Innsbruck Austria; ^2^ Department of Botany and Biodiversity Research University of Vienna Vienna Austria; ^3^ Department of Evolutionary Biology, Ecology and Environmental Sciences University of Barcelona Barcelona Spain

**Keywords:** bedrock, comparative phylogeography, genetic structure, Pyrenees, RADseq, species distribution models

## Abstract

**Aim:**

Species' ecological traits influence their spatial genetic patterns. Bedrock preference strongly shapes the phylogeography of alpine plants, but its interactions with other ecological traits have rarely been disentangled. Here, we explore whether dispersal ability and degree of habitat specialization account for divergent postglacial expansion patterns of high‐elevation plants in spite of similar bedrock preference.

**Location:**

The Pyrenees, southwestern Europe.

**Taxon:**

*Cirsium glabrum* (Asteraceae), *Salix pyrenaica* (Salicaceae) and *Silene borderei* (Caryophyllaceae).

**Methods:**

Phylogenetic, genetic structure and demographic modelling analyses based on restriction‐site‐associated DNA sequencing (RADseq) data from a range‐wide populational sampling were conducted. Occurrence data and environmental variables were used to construct species distribution models, which were projected under current and Last Glacial Maximum conditions, and were combined with RADseq data to reconstruct the postglacial history of the study species. The degree of habitat specialization of each species was estimated based on the plant communities within which they occur, and their climatic niche breadth.

**Results:**

*Salix pyrenaica*, which occupies a broad range of habitats, shows a high level of range filling, a blurred genetic structure and an admixture cline between the two main genetic groups, congruent with rapid postglacial expansion. The microsite specialists *C. glabrum* and *S. borderei* exhibit a strong genetic structure and low levels of range filling, indicative of slow postglacial expansion. The good disperser *C. glabrum* shows higher levels of admixture between genetic groups and weaker population differentiation than the poor disperser *S. borderei*.

**Main Conclusions:**

Factors other than bedrock preference have a strong impact on the postglacial range dynamics of high‐elevation species. Habitat specialization plays an important role, allowing species occupying a broad range of habitats to more rapidly expand their ranges after environmental change. The effect of dispersal ability is lower than expected for the study species.

## 1 INTRODUCTION

The genetic structure of species is a direct reflection of their evolutionary histories, which are, in turn, strongly affected by biotic and abiotic factors (Avise, 2000; Hewitt, 2000). Because of its strong influence on the chemical properties of soils, bedrock type is a factor of major importance driving the distribution of plant species (Ellenberg & Leuschner, 2010). In geologically heterogeneous areas, patches of a specific bedrock composition likely act as islands or barriers for plants requiring particular soil conditions. Specifically, the degree of isolation and the size of these patches strongly determine demographic parameters of populations, such as effective population size or the frequency and source of migrants, thus shaping the spatial genetic structure of species.

A further decisive factor shaping the distributions of species and their spatial genetic patterns is climate (e.g. Forster, 1778; Pearson & Dawson, 2003). Changes in climate therefore strongly impact the spatial genetic structure and intraspecific diversity of species, especially where these changes have enforced massive range shifts. Both bedrock and climatic oscillations are expected to have been of particular importance for shaping the current distribution of the flora of temperate mountain regions such as the Alps or the Pyrenees, where Pleistocene glaciations were particularly strong (Schönswetter et al., 2005), the tectonic structure is complex, and the commonly thin soils in high elevations are only weakly buffered against the chemistry of the bedrock (Alvarez et al., 2009).

However, alongside bedrock and climate, ecological traits of the species such as habitat specialization and dispersal ability strongly impact their distribution and spatial genetic structure (Davis et al., 1998; Lawton, 2000; Wright, 1943). These features interact and are major drivers of effective dispersal, that is, the foundation of new populations, a crucial feature in range expansions in a heterogeneous landscape (Clark et al., 2007; Gillespie et al., 2012; MacArthur & Wilson, 1967; Silvertown, 2004). For example, species occupying a narrow range of habitats (termed ‘microsite specialists’ in the following) and poorly adapted to long‐distance dispersal will experience low rates of effective dispersal, resulting in a lower ability to spatially track climatic oscillations (postglacial migration lag; Dullinger et al., 2012; Kropf et al., 2002; Schneeweiss & Schönswetter, 2010; Svenning et al., 2008). In contrast, species occupying a broad range of habitats and with good dispersal abilities will be able to better track climatic oscillations with faster distribution shifts (distribution equilibrium; Araújo & Pearson, 2005; Dullinger et al., 2012). These differential species‐specific abilities, together with other demographic history parameters such as effective population size or the duration of divergent evolution between species (Marko & Hart, 2011), will result in an array of idiosyncratic spatial genetic patterns, even for co‐distributed species with similar bedrock preferences. This could explain why pervasive phylogeographical patterns are rarely detected in comparative phylogeographical studies (Brunsfeld et al., 2001; Dawson, 2014; Kirschner et al., 2020; Papadopoulou & Knowles, 2015).

**FIGURE 1:**
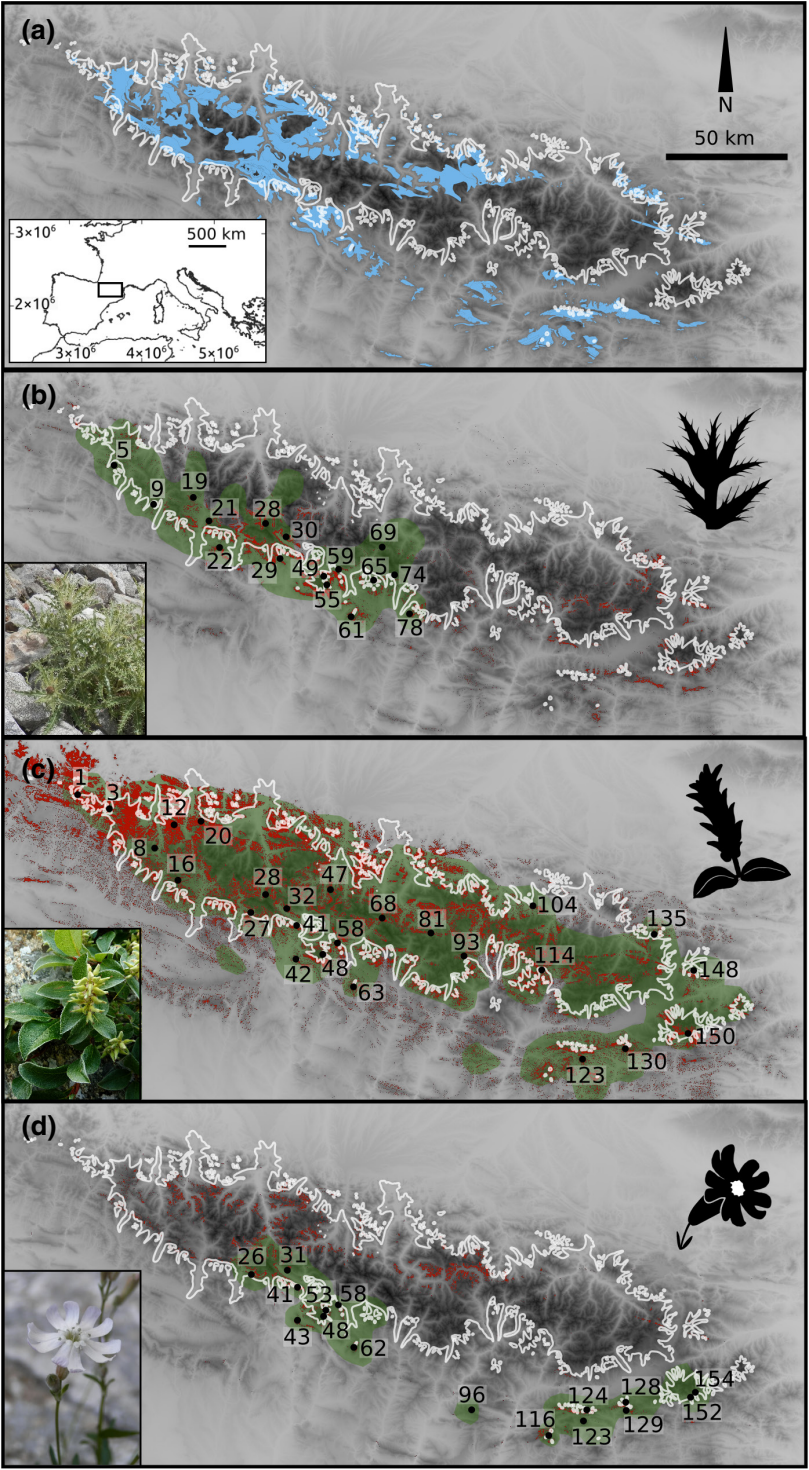
(a) Study area, maximum extent of glaciers at the Last Glacial Maximum (white line) and areas with carbonate bedrock above 1500 m (blue). (b)–(d) Distribution (green), collected populations (numbered) and predicted suitable areas under current climatic conditions (red) of *Cirsium glabrum* (b), *Salix pyrenaica* (c) and *Silene borderei* (d). Maps generated with Lambert azimuthal equal area projection, with datum ETRS89. Photos in (b) and (d) by Pau Carnicero, (c) by I. Blanc (https://creativecommons.org/licenses/by‐sa/4.0/deed.en)

The combined effects of bedrock, climatic oscillations and species traits on spatial genetic patterns are ideally studied in mountain systems, which are geologically heterogeneous and strongly affected by Pleistocene glaciations. The Pyrenees are a southern European mountain range with a geological origin dating to the Hercynian orogeny (ca. 200–300 Ma), while the main uplift occurred during the Alpine orogeny (37–24 Ma). The main chain is mostly built of granite and metamorphic rocks of siliceous nature, flanked by carbonate‐rich sedimentary rocks. However, this large‐scale pattern is interrupted by several islands of carbonate‐rich substrates along the main chain, as well as silicate‐rich sediments in the Pre‐Pyrenees, resulting in a geologically heterogeneous landscape. The typical postglacial landscape, with U‐shaped valleys, moraines and glacial cirques, evidences the extensive glaciation during the cold stages of the Pleistocene (Figure 1, Calvet, 2004; Calvet et al., 2011). However, the eastern and western margins of the main chain, the southern ranges of the Pre‐Pyrenees, as well as most crests and summits remained largely ice free (Arribas, 2004), and probably acted as glacial refugia for high‐elevation species.

Here, we used phylogeographical analyses based on genomic data and species distribution modelling to study the genetic structure and diversity as well as the environmental niche of three calcicolous species with different dispersal abilities and degree of habitat specialization within the diversity of calcareous habitats. In particular, we explored the relative impact of three factors, that is bedrock preference, habitat specialization and dispersal ability, on species' current spatial genetic patterns. Specifically, if bedrock alone is a good predictor of spatial genetic patterns, we expect to find congruent phylogeographical patterns across the three species. However, if dispersal ability and degree of habitat specialization had a strong impact on the range contraction to glacial refugia and posterior expansion strategies, we expect species‐specific spatial genetic patterns and differential degrees of range filling. Specifically, we expect to find a blurred genetic structure and a high degree of range filling for good dispersers and species occupying a broad range of habitats, and a strong genetic structure and incomplete range filling for poor dispersers and microsite specialists. Furthermore, we aimed at disentangling the contribution of habitat specialization and dispersal ability to the species' postglacial range expansion. The three studied species are *Salix pyrenaica*, a species occupying a broad range of habitats with good dispersal ability, *Cirsium glabrum*, a microsite specialist with good dispersal ability, and *Silene borderei*, a microsite specialist with low dispersal ability. All of them are Pyrenean endemics, which allows ruling out recent immigration or gene flow from neighbouring mountain ranges.

## 2 MATERIALS AND METHODS

### 2.1 Study species

The three studied species are Pyrenean endemics with preference for carbonate bedrock, including limestone and other carbonate sediments as well as schists. They all occur at elevations spanning the subalpine and alpine belts, that is, ca. (1000) 1500–2800 m. *Cirsium glabrum* is a herbaceous geophyte with a subterranean rhizome (Talavera, 2014). It grows on steep slopes formed by limestone and schist screes in the central and western Pyrenees. The species reproduces sexually, and has potentially good anemochorous dispersal abilities due to the high number of fruits per head and the presence of a dispersal appendage (pappus). Although it is mostly found at the subalpine belt, it can reach down to 1000 m. *Salix pyrenaica* is a prostrate shrub reaching a maximum height of ca. 50 cm (Blanco, 2005). It occurs in a diversity of rocky habitats from crests to stony meadows, usually in places with long snow cover, across the entire mountain range. It is dioecious, with inconspicuous flowers and hairy seeds well adapted to anemochorous dispersal. Finally, *S. borderei* is a perennial herb with slightly woody ground shoots (Talavera, 1990). It is a weak competitor growing exclusively in crevices of vertical to overhanging shady limestone cliffs. It occurs mostly in the southern Pre‐Pyrenees, with occasional occurrences on limestone outcrops along the main chain. The seeds are small and have no apparent adaptations to dispersal over long distances. The dispersal abilities of the study species have been inferred according to the above‐mentioned traits and published dispersal kernels (Bullock et al., 2017).

### 2.2 Plant material

**FIGURE 2:**
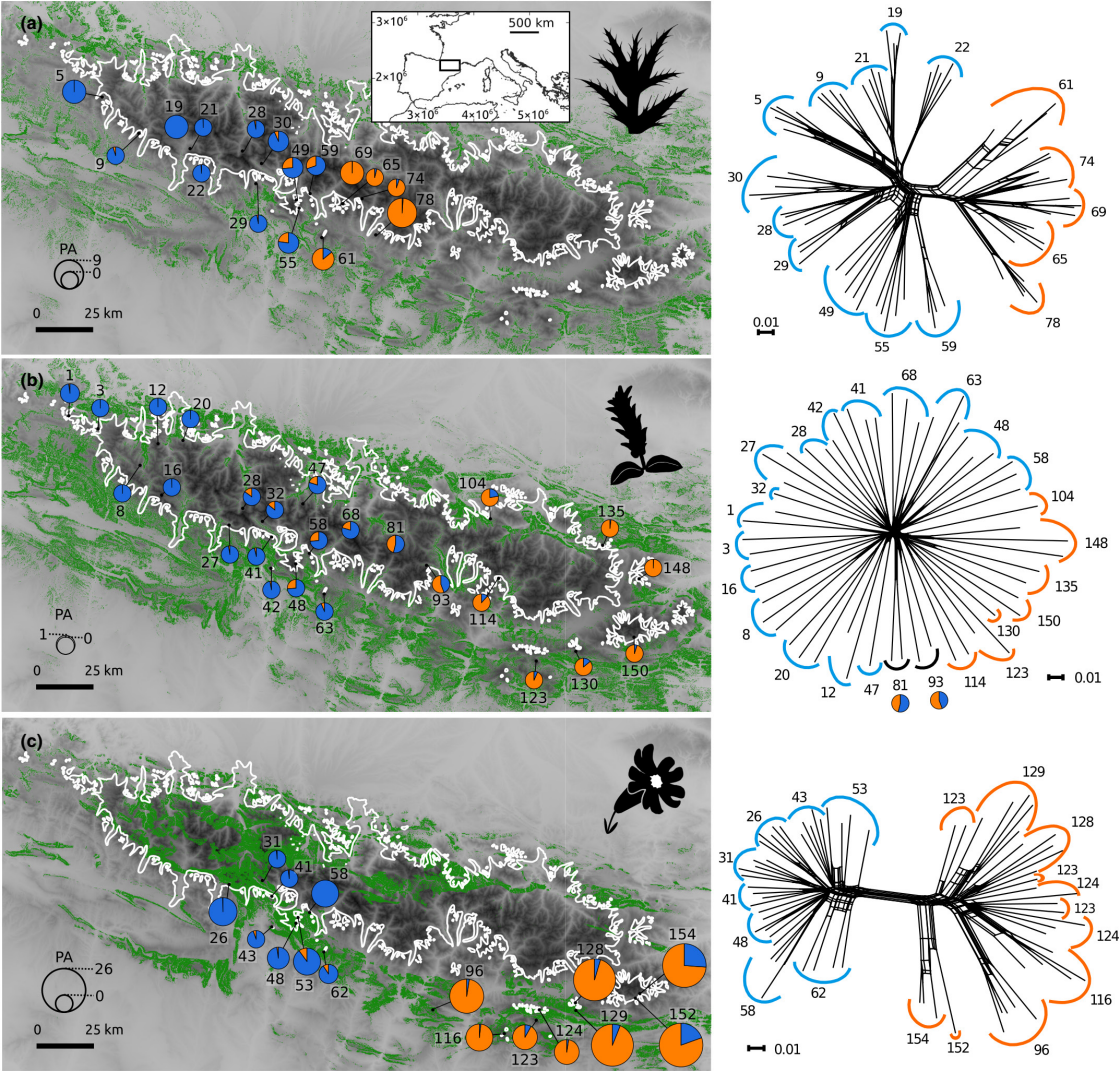
Genetic structure of the study species. Left panels: Pie charts indicate the admixture proportions at *K* = 2 derived from STRUCTURE analyses. The size of the pie charts reflects the number of private alleles (see scale in the lower left corner). The white line indicates the maximum extent of glaciers during the Last Glacial Maximum (LGM), green shaded areas represent suitable areas at LGM conditions. Right panels: Neighbour‐nets, each population is marked with colours according to the most probable genetic group in STRUCTURE analyses. Populations with <70% assignment to a group are in black, and admixture pie charts are presented. (a) *Cirsium glabrum*, (b) *Salix pyrenaica*, (c) *Silene borderei*. Maps generated with Lambert azimuthal equal area projection, with datum ETRS89

Samples of the study species were collected from 52 sites in 2018 covering the entire distribution range of each species (Figure 2; Table S1). Sites (in the following termed ‘population’ for simplicity) were numbered from west to east, preceded by a species acronym (Cg, Sp and Sb). Young and healthy leaves of three (Sp) to five individuals (Cg, Sb) per population were sampled and stored in silica gel for the extraction of DNA and flow cytometry. In cases of small populations (Sb152, Sb154), fewer individuals were sampled to avoid threats to population survival. In the case of *C. glabrum* and *S. pyrenaica*, sampling was conducted on individuals spaced at least 5 m to avoid sampling clones. From each population, a herbarium voucher was made and deposited in the herbarium of the University of Barcelona (acronym BCN). Sampling was carried out in accordance with the Nagoya Protocol, and the EU regulation 511/2014, that is, prior informed consent was requested from the competent national authorities of Nagoya protocol members (https://www.cbd.int/abs/about) and a collection permission was obtained if required. *Cirsium erisithales* Scop., *C. spinosissimum* Scop., *Salix hastata* L., *S. lanata* L., *Silene ciliata* Pourr., *S. multicaulis* Durand. and *S. saxifraga* L. were sampled as outgroups.

### 2.3 Flow cytometry

Relative genome size (RGS) measurements were conducted with leaf material from all collected populations, aiming at checking for uniform ploidy level across populations for each species (Table S1). Three individuals per population were measured in case of *S. pyrenaica*, two for *S. borderei* and one for *C. glabrum*. These different sampling sizes are due to difficulties in obtaining high‐quality estimates for *C. glabrum* and *S. borderei*. No results were obtained for populations Cg62, Cg65, Sb152 and Sb154. Flow cytometry of 4′,6‐diamidino2‐phenylindole stained nuclei was used to estimate RGS of the dried samples. *Bellis perennis* L. (2C = 3.38 pg; Schönswetter et al., 2007), growing in the Botanical Garden of the University of Innsbruck, was used as standard. Dried leaf tissue was chopped together with fresh leaf material of the standard and processed as described in Suda et al. (2007). The relative fluorescence intensity of 3000 particles was recorded using a CyFlow Space flow cytometer (Sysmex). Partec FloMax software was used to evaluate the histograms. RGS was calculated as the ratio between the relative fluorescence of sample and standard. The reliability of the measurements was assessed by calculating coefficients of variation (CV) for the G1 peaks of both the analysed sample and the standard. Analyses yielding a CV threshold of >5% were discarded and the samples measured again, except for *C. glabrum*, for which it was in some cases not possible to obtain better estimates.

### 2.4 DNA extraction

Total genomic DNA was extracted from ca. 10 to 20 mg dried leaf material following the CTAB protocol (Doyle & Doyle, 1987) with some modifications (Tel‐Zur et al., 1999). The ground leaf material was washed once for *Salix*, twice for *Silene* and three times for *Cirsium* samples with the wash buffer containing sorbitol. The quality of the extracts was examined photometrically. Extractions were then purified with the Nucleospin gDNA clean‐up kit. The DNA concentration was estimated using a Qubit 4 fluorometer (ThermoFisher Scientific).

### 2.5 RADseq: Library preparation, identification of RADseq loci and single nucleotide polymorphism calling

Single‐digest restriction‐site‐associated DNA sequencing (RADseq) libraries were prepared from five individuals per each *C. glabrum* and *S. borderei* population, three individuals per each *S. pyrenaica* population and at least one individual of each outgroup species (Table S1) using the restriction enzyme *Pst*I (New England Biolabs) and a protocol adapted from Paun et al. (2016). Briefly, we started with 100–140 ng DNA per individual and ligated 100 mm P1 adapters to the restricted samples. Shearing by sonication was performed with a M220 Focused‐ultrasonicator (Covaris) with settings targeting a size range of 200–800 bp and a mode at 400 bp (peak in power: 50, duty factor 10%, 200 cycles per burst and treatment time 90 s at 20°C). Libraries were sequenced on Illumina HiSeq at VBCF NGS Unit (http://www.vbcf.ac.at/ngs/) as 100 bp single‐end reads.

The raw reads were quality filtered and demultiplexed based on individual‐specific barcodes using Picard BamIndexDecoder included in the Picard Illumina2bam package (available from https://github.com/wtsinpg/illumina2bam) and the program process_radtags.pl implemented in Stacks 2.3 (Catchen et al., 2011, 2013). The RADseq loci were further assembled, and single nucleotide polymorphisms (SNPs) were called using the ‘denovo_map.pl’ pipeline as implemented in Stacks. To select the optimal parameters, a preliminary optimization step following the 80% rule (Paris et al., 2017) was conducted. First, we selected 12–15 samples of each species and ran denovomap.pl for different values of the number of mismatches allowed between stacks to merge them into a putative locus (*M*) from 0 to 8 and a percentage of individuals that must possess a particular locus for it to be included in the calculation of population‐level statistics (*r*) of 80%. For every value of *M*, the minimum number of raw reads required to form a stack (*m*) and the maximum number of differences among loci to be considered as orthologous across multiple samples (*n*) were given values equal to *M*. After the optimization, *M* = 5 was selected as the value optimizing the number of de novo assembled loci for the three species. The values of *m* and *n* were as well set to 5.

The program Populations implemented in Stacks was used to export the selected loci and generate population genetics statistics. Preliminary exploratory analyses with the R package ‘Adegenet 2.1.3’ (https://CRAN.R‐project.org/package=adegenet) on files generated under different filtering parameters allowed selecting a filtering scheme with a good balance of missing data and amount of informative characters. Samples with high levels of missing data were excluded (Table S2) and the program Populations was run again for the cropped dataset. The vcf files were further filtered with VCFtools 0.1.16 (Danecek et al., 2011). The filters applied in every analyses can be found in Table S2.

### 2.6 Exploratory analyses of SNP data

Adegenet was used to calculate Nei's distance matrices (Nei, 1972), which were used to construct a neighbour‐net (Bryant & Moulton, 2004) in SplitsTree 4.14.2 (Huson & Bryant, 2006). To infer phylogenetic relationships among individuals of each species, we computed maximum likelihood (ML) phylogenetic analyses using RAxML 8.2.12 (Stamatakis, 2014). Invariant sites were removed from the original phylip format using the script ‘deleteAlignColumn.pl’ (available from https://www.biostars.org/p/55555/) and Felsenstein's ascertainment bias correction was further used to account for missing invariant sites as recommended by Leaché et al. (2015). Tree searches were done under a General Time Reversible model with disabled rate heterogeneity among sites as recommended in the manual (ASC_GTRCAT; ‐V; Stamatakis, 2014). The best‐scoring ML tree was bootstrapped using 1000 replicates and the frequency‐based stopping criterion (Pattengale et al., 2010). The results were visualized with FigTree 1.4 (available from http://tree.bio.ed.ac.uk/software/figtree/; last accessed January 27, 2021).

The optimal grouping of individuals was determined using Bayesian clustering in STRUCTURE 2.3.4, using the admixture model with uncorrelated allele frequencies (Pritchard et al., 2000). To meet the assumption of unlinked SNPs only the first SNP per locus was kept (‐‐write_single_snp). Ten replicate runs for *K* (number of groups) ranging from 1 to 10 were carried out using a burn‐in of 50,000 iterations followed by 500,000 additional MCMC iterations. CLUMPAK (Kopelman et al., 2015) was used to summarize the results across different *K* values and to produce plots. The optimal *K* was identified as the *K* where the increase in likelihood started to flatten out, the results of replicate runs were similar and the clusters were non‐empty. Additionally, the deltaK criterion was employed, reflecting an abrupt change in likelihood of runs at different *K* (Evanno et al., 2005). Additionally, fineRADstructure (Malinsky et al., 2018) was used to infer the coancestry matrix of the same dataset. We used the program Populations in Stacks to estimate several summary statistics, such as the number of private alleles, the nucleotide diversity (*π*) per population and genetic group and *F*_ST_ between genetic groups. For the estimation of population‐level summary statistics, geographically close populations of *C. glabrum* and *S. borderei* were defined as single populations (Table S1).

### 2.7 Demographic modelling

To explore possible population size changes associated with the last glaciation, we modelled the effective population size (*Ne*) using the software Stairway plot (Liu & Fu, 2015). First, we computed the folded site frequency spectrum (SFS) of each species and genetic group using easySFS (https://github.com/isaacovercast/easySFS) by downprojecting the datasets to a minimum sample size, which maximizes the number of SNPs kept (Table S2; Gutenkunst et al., 2009). Individuals with <70% assignment to a genetic group in the STRUCTURE analysis were excluded for the calculation of population‐specific SFSs. We ran Stairway plot on 200 bootstrap replicates drawn from the calculated SFSs. The median *Ne* and confidence intervals were obtained based on 200 estimations. In the absence of reliable data for closely related species, we used the mutation rate of *Arabidopsis thaliana* (L.) Heynh. (7 × 10^−9^, Ossowski et al., 2010) and allowed a deviation of 10% from the above rate to account for uncertainty. No information on the generation times for the study species is available, but it is known that dwarf‐shrubs such as *S. pyrenaica* need a longer time span until the first reproductive event than perennial herbs (Körner, 2003). Therefore, and to represent a reasonable range of genealogical scenarios, three different generation times per species were used: 5, 10 and 20 years for herbaceous *C. glabrum* and *S. borderei*, and 10, 20 and 30 years for woody *S. pyrenaica*.

To explore alternative demographic models and explore potential differences among the demographic histories of the study species, we used the diffusion approximation method of dadi (Gutenkunst et al., 2009) to analyse joint site frequency spectra (JSFS). We fitted two‐population demographic models using dadi_pipeline 3.1.4 (Portik et al., 2017). Given the nested patterns observed in the phylogenetic trees of *C. glabrum* and *S. borderei* (see Section  3), we ran the 14 island diversification models—that is, models in which one population originates from a founder event—for these two species (Charles et al., 2018), defining the western population as ancestral for *C. glabrum*, and the eastern as ancestral for *S. borderei*. The standard diversification models—that is, vicariance models—were explored for *S. pyrenaica*, excluding models with three demographic epoches after population divergence, to avoid excessive complexity (Charles et al., 2018; Portik et al., 2017). For all models, we performed consecutive rounds of optimizations. For each round, we ran multiple replicates and used parameter estimates from the best scoring replicate (highest log‐likelihood) to seed searches in the following round. We used the default settings in the dadi_pipeline for each round (replicates = 10, 20, 30, 60; maxiter = 3, 5, 10, 15; fold = 3, 2, 2, 1), and optimized parameters using the Nelder–Mead method (optimize_log_fmin). Across all analyses, we used the optimized parameter sets of each replicate to simulate the 2D‐JSFS, and the multinomial approach was used to estimate the log‐likelihood of the 2D‐JSFS given the model. Finally, the inferred parameters of the models were converted from genetic units to conventional units (years and number or proportion of individuals; Gutenkunst et al., 2009).

### 2.8 Environmental variables

To account for the rough terrain of mountain regions, the available historic climate dataset, accessed via PaleoView (Fordham et al., 2017) with a coarse spatial resolution of 2.5°, as well as current climate data retrieved from the Chelsa Climate database (Karger et al., 2017) available at http://chelsa‐climate.org/ were statistically downscaled to 100 m resolution using the ‘delta‐method’ (see details in Appendix S5). Here, two reference periods were used, the Last Glacial Maximum (LGM, i.e. 30 years centred around 21,000 BP) and recent climatic conditions (i.e. 1979–2013). We used the downscaled monthly projections of climate variables to calculate two bioclimatic variables representing climatic conditions during the growing season: mean temperature of the warmest quarter (bio10) and precipitation sum of the warmest quarter (bio18). We finally used a digital elevation model (DEM, obtained from https://www.eea.europa.eu/data‐and‐maps/data/copernicus‐land‐monitoring‐service‐eu‐dem) to calculate slope inclination. These variables were checked to exclude high correlations (Pearson's *r* > |0.7|).

Bedrock was extracted from the geological map of the Pyrenees (Anonymous, 2009; obtained from http://info.igme.es/cartografiadigital/geologica/mapa.aspx?parent=../tematica/tematicossingulares.aspx&Id=14&language=es). We classified the geological entities into four categories reflecting the amount of carbonate, from pure silicate to pure carbonates (Table S3).

### 2.9 Modelling occurrence probability

We used species distribution models (SDMs) to project the environmental suitability of the study area for the three studied species at the LGM and under current conditions. Species occurrence data were obtained from our sampling sites and GPS data obtained during field work, and a selection of precise localities from the databases of the Atlas of the Flora of the Pyrenees (Gómez‐García, 2014; (http://161.116.68.78/florapyrenaea/) and Silene (http://flore.silene.eu), resulting in 38 data points for *C. glabrum*, 27 for *S. borderei* and 137 for *S. pyrenaica*. Absences were generated from a dataset comprising 2229 non‐forested vegetation plots above 1000 m, georeferenced at a precision of 1 × 1 km and containing at least information on elevation from the European vegetation archive (EVA; Chytrý et al., 2016) and the Iberian and Macaronesian vegetation information system (Font et al., 2017). To more accurately extract the precise climatic conditions of these plots, we used elevation, aspect and slope to select topographically matching 100 × 100 m cells within 1 km distance of the sampled plot (altitude ±100 m, slope ±10° of plot value, aspect ±36° of plot value), following the approach of Dullinger et al. (2012). The values of the environmental variables were then averaged for each point across the selected 100 × 100 m cells. We finally used 1247 absence data points for *C. glabrum* and *S. borderei* and 1207 for *S. pyrenaica*.

Species distribution models were parameterized using the R‐package ‘biomod2’ (Thuiller et al., 2019) by correlating species presence‐absence data to the environmental variables by means of five modelling techniques, that is generalized linear models (GLM), generalized additive models (GAM), boosted regression trees (GBM), random forests (RF) and maximum entropy (MAXENT). To evaluate model quality for each species and modelling technique, the data were randomly split into one subset for calibrating the models (80%) and another one for evaluating them (remaining 20%) using True Skill Statistic scores (TSS, Allouche et al., 2006). This process was repeated 10 times to make sure that the estimated predictive accuracy was not influenced by the random partitioning. Only techniques with a mean TSS ≥0.7 were used to construct final models, which were calibrated with 100% of the data to avoid loss of information for species represented by a low number of occurrences only. The probability of occurrence for each 100 × 100 m cell under current and LGM climatic conditions was then computed as the mean of projections of the used modelling techniques for each species (Araújo & New, 2007). Binary presence–absence maps were produced using TSS to find the optimal threshold. In the case of *S. pyrenaica*, a clamping mask based on a model without bedrock information was created, as biomod cannot create a clamping mask in presence of categorical variables. This layer was then used to exclude those areas in the projected layers where one or more variables were out of the predictor variables range, as the response of species under such conditions is unknown (Elith et al., 2011).

### 2.10 Estimates of habitat specificity and niche breadth

To estimate the degree of specialization of the studied species, vegetation data from EVA (Chytrý et al., 2016) were used to estimate the amount of variability in the species composition of communities where the studied species were recorded. In total, 49 vegetation surveys for *C. glabrum*, 12 for *S. borderei* and 174 for *S. pyrenaica* constituted the dataset. An ordination analysis (detrended correspondence analysis) using the R‐package ‘vegan’ (Oksanen et al., 2020) was performed. Additionally, niche breadth and overlap were calculated from the presence data and predictor variables used for the SDMs by means of dynamic range boxes (Junker et al., 2016) implemented in the R‐package ‘dynRB’ (Schreyer et al., 2021).

## 3 RESULTS

### 3.1 Population structure and phylogenetic relationships

The average number of high‐quality reads per sample retained after demultiplexing and quality filtering was 0.88 (SD = 0.16) million. The resulting number of SNPs per species and filtering scheme is presented in Table S2.

**FIGURE 3:**
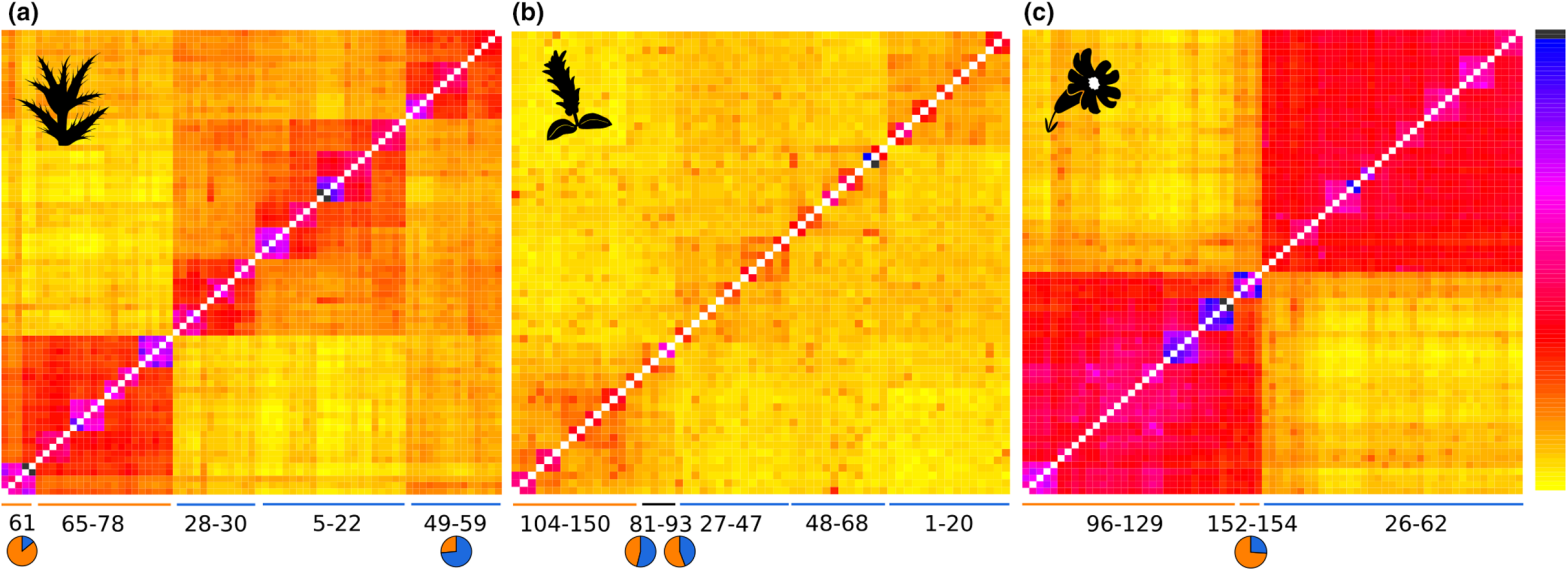
FineRADstructure coancestry matrices of the study species. Blue indicates maximum levels of coancestry between two individuals, yellow the minimum (scale on the right). Numbers below the plots indicate the population numbers and the coloured lines correspond to the most probable genetic group in STRUCTURE analyses at *K* = 2. Admixture pie charts from STRUCTURE analyses are presented for populations with high admixture levels discussed in the text. (a) *Cirsium glabrum*, (b) *Salix pyrenaica*, (c) *Silene borderei*

The neighbour‐net of *C. glabrum* showed two clear groups, the eastern group comprising five populations and the western group 11 populations (Figure 2). The RAxML phylogenetic tree rooted with the outgroup resulted in a monophyletic eastern group (Figure S1, BS 100%), sister with low support to the easternmost populations of the western group (BS 67%), and nested in a clade with several subclades corresponding to geographical groups with unresolved relationships. With two exceptions (populations 74 and 49), all populations constituted monophyletic groups. STRUCTURE resolved two groups corresponding to the above mentioned (Figure 2). However, admixture was detected (22%–33%) in the easternmost populations 49, 55 and 59 of the western group and in two individuals of the eastern population 61 (18% and 30%). At *K* = 3, the western group is split in two groups linked by an east–west gradual admixture cline (Figure S2). The fineRADstructure results showed similar results, with a well‐defined, uniform eastern group, and a more structured western group, reflecting the above‐mentioned admixture signal between the two groups (Figure 3). Summary statistics at the group and the population levels for each of the three species are presented in Table 1 and Table S1, respectively.

**TABLE 1:** Population genetics summary statistics calculated for the two main groups detected with STRUCTURE (E, eastern group; W, western group)

	*F*_ST_ E – W	PA – W	PA – E	*π* – W	*π* – E
*Cirsium glabrum*	0.753	1034	201	0.284	0.242
*Silene borderei*	0.116	1292	2056	0.219	0.263
*Salix pyrenaica*	0.021	2198	71	0.288	0.284

*Note*: PA, number of private alleles; *π*, nucleotide diversity.

A marked geographical structure can be observed in *S. borderei* in the neighbour‐net, with the main east–west differentiation consistent with a broad gap in the species' distribution in the central Pyrenees (Figure 2). The eastern group showed a certain degree of substructure, with the easternmost populations (152 and 154) clustering separately from the rest of the eastern populations in the neighbour‐net. These two populations constitute a clade (99%) sister to the remaining populations (89%) in the RAxML tree (Figure S3). The remaining eastern populations and all western populations form two sister clades with maximum support (100%). Most populations constituted monophyletic groups; only two (123 and 124) had individuals allocated to different, statistically supported clades. The STRUCTURE results at *K* = 2 retrieved the same two groups, with some admixture in the easternmost populations (152 and 154; 20%–22%). The same pattern was observed in fineRADstructure results, with additional traces of admixture between populations adjacent to the gap between the western and eastern groups (62 and 96, Figure 3). The STRUCTURE results at *K* = 3 resulted in an additional group for the westernmost population of the eastern group (96; Figure S2). The substructure in each group was explored with STRUCTURE and fineRADstructure. STRUCTURE showed four geographically correlated subgroups within the eastern group, which could also be observed in the fineRADstructure matrix (Figure S4). Substructure within the western group was less clear (not shown).

The neighbour‐net of *S. pyrenaica* was star‐like, with weak geographical structure between the eastern and western parts of the distribution. The RAxML tree showed mostly unsupported relationships (Figure S5). At *K* = 2, the best clustering scheme in STRUCTURE, an eastern and a western group were resolved, which border in the central Pyrenees; two populations in this area were strongly admixed. The populations of each group showed a gradual increase of admixture towards the border between the two groups, non‐admixed populations were restricted to the eastern‐ and westernmost extremes of the distribution. FineRADstructure showed a very shallow structure, with a slightly better defined eastern group, while the western group could not be identified (Figure 3).

### 3.2 Demographic modelling

**FIGURE 4:**
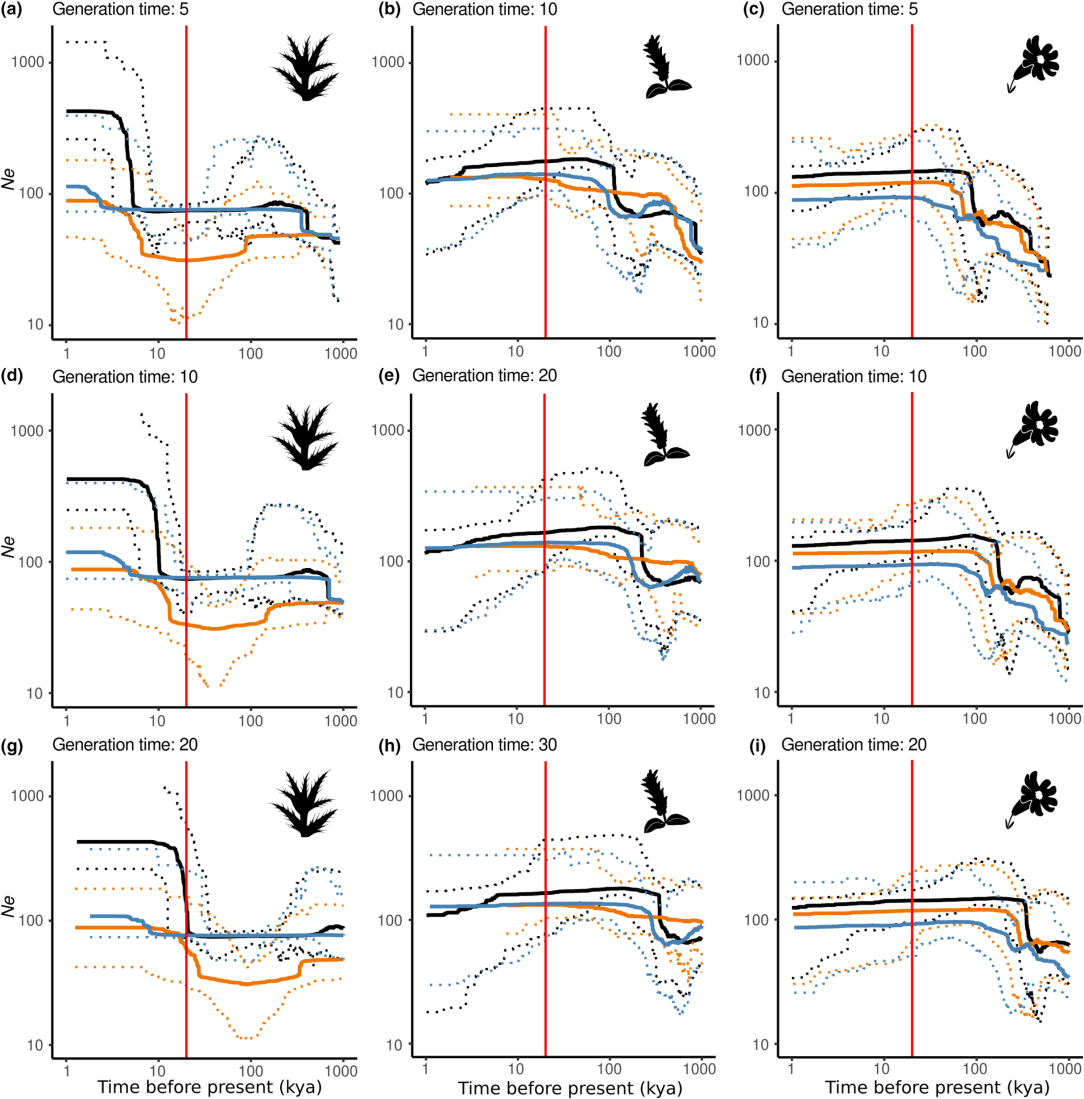
Changes in effective population size of the study species estimated with stairway plots for different generation time values. Coloured lines indicate the genetic groups from STRUCTURE analyses at *K* = 2, black lines correspond to entire species. Dashed lines indicate the 95% confidence intervals of the median *Ne*. The dotted red vertical line indicates the Last Glacial Maximum. (a, d, g) *Cirsium glabrum*, (b, e, h) *Salix pyrenaica*, (c, f, i) *Silene borderei*

The stairway plot demographic models conducted on the three species showed past increases in *Ne*, albeit at different periods and with different magnitudes (Figure 4). At the species level, *C. glabrum* showed a strong increase in population size at ~5–10 ka, which can be observed within the eastern group as well. The western group showed a moderate increase at ~3–5 ka. In both *S. borderei* and *S. pyrenaica*, increases in population size were observed much earlier, at ~200–500 ka. In *S. borderei*, the increase was consistent at the species level and within the eastern and western groups, though less marked for the western group. Finally, for *S. pyrenaica*, an increase was only observed at the species level and for the western group. The current *Ne* values for mid values of generation time and mutation rate are 426,000 for *C. glabrum*, 126,000 for *S. borderei* and 106,000 for *S. pyrenaica*. The degree of uncertainty introduced using different mutation rates and generation times did not qualitatively change the interpretation of the results (Figure 4, Figure S6).

The best‐supported two‐populations demographic models for *C. glabrum* (Akaike information criterion [ΔAIC] = 106) and *S. borderei* (ΔAIC = 86.8) involved a founder event plus asymmetric migration, in both cases higher from the source population to the founded population than vice versa (m21 > m12, Figure S6, Table S4). In *S. pyrenaica* the most likely model was a two‐epoch model with asymmetric migration (ΔAIC = 21.8), with 10‐fold higher migration from the eastern to the western population than vice versa (Figure S7, Table S4). Population size increases were inferred in both populations. We emphasize that here we do not focus on the exact parameter estimates, but, rather, on the generalities of the models inferred. Reliable parameter estimates should be estimated and bootstrapped in a coalescent simulator (Gutenkunst et al., 2009). However, inferred parameters are presented to allow for comparison among species in Table S4.

### 3.3 Modelling occurrence probability

The TSS of the ensemble models of the three species were >0.9, indicating a high predictive accuracy (Table S2). The actual distribution areas of the species are in general well covered by the SDM predictions for the current climatic conditions (Figure 1), although some areas predicted as suitable are not occupied by the species. The biggest predicted areas for *C. glabrum* are located along the southern margin of the Pre‐Pyrenees, at the centre of its current distribution. However, additional highly suitable areas are predicted in the eastern Pyrenees, where the species does not occur. In the case of *S. pyrenaica*, the prediction exceeds the current distribution towards the west. The predicted highly suitable areas of *S. borderei* are overrepresented, especially in western and central Pyrenees well within the LGM ice sheet. In general, occurrence probability for the studied species at the LGM shifted towards the northern and southern margins of the glaciers for *C. glabrum* and *S. pyrenaica*, while for *S. borderei* several of the predicted areas under the ice sheet increased strongly (Figure 2).

### 3.4 Estimates of habitat specificity and niche breadth

**FIGURE 5:**
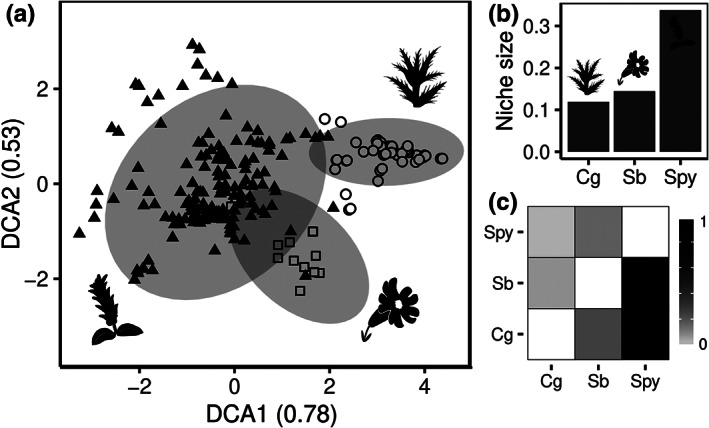
Ordination of vegetation data and niche breadth analyses of the study species. (a) Detrended correspondence analysis of vegetation relevés containing the three study species. Circles correspond to relevés with *Cirsium glabrum*, triangles to relevés with *S. pyrenaica* and squares to relevés with *Silene borderei*. Shaded ellipses indicate 99% confidence intervals. (b) Niche size of the study species. (c) Niche overlap between pairs of study species. The grey scale indicates the proportion of the niche of the species along the y‐axis contained in the niche of the species along the x‐axis. The cells along the diagonal are empty. In (b) and (c), calculations were performed using dynamic range boxes (Junker et al., 2016), species names were abbreviated. Cg, *C. glabrum*; Spy, *S. pyrenaica*; Sb, *S. borderei*

*Salix pyrenaica* occurs in a wide diversity of plant communities, whereas *C. glabrum* and *S. borderei* occur in less divergent plant communities, as indicated by their more closely grouped points in the DCA (Figure 5a). In the same line, the estimated niche size based on predictor variables showed an about three times broader niche for *S. pyrenaica* (0.34) than that of *C. glabrum* (0.12) and *S. borderei* (0.14) (Figure 5b). The results of the overlap analyses showed that the niches of *C. glabrum* and *S. borderei* are to a big extent contained in the niche of *S. pyrenaica*, but not vice versa (Figure 5c).

## 4 DISCUSSION

Our results demonstrate two main responses of high‐elevation plant species of the Pyrenees to the temperature increase at the end of the last glacial period, that is on the one hand a rapid range expansion towards distribution equilibrium in *S. pyrenaica*, and, on the other hand, postglacial migration lag in *C. glabrum* and *S. borderei*. A strong source of evidence comes from the species' level of range filling (Dullinger et al., 2012; Svenning & Skov, 2004), that is, the degree to which the species distribution fills the SDM predictions under current conditions (Figure 1). The almost complete overlap between modelled and actual distributions observed for *S. pyrenaica* indicates successful postglacial colonization of areas, which were glaciated or non‐suitable at the LGM, from peripheral refugia (Figure 2). Nunatak survival can be ruled out as a main explanation for this rapid colonization as the few predicted suitable patches within the LGM ice sheet are situated in valleys, and were thus covered by glaciers (Figure 2). In contrast, the current distributions of *C. glabrum* and *S. borderei* cover only a small fraction of their potential range (Figure 1), most often corresponding to areas located outside of the LGM glaciers or close to their southern margins, while suitable areas close to their current distribution but within the LGM glaciers remain unoccupied. A postglacial migration lag has frequently been reported for alpine plants (Dullinger et al., 2012; Kropf et al., 2002; Schneeweiss & Schönswetter, 2010; Svenning et al., 2008), whereas other studies have suggested a high capability of mountain plants to track environmental changes (Engler et al., 2009).

The increase in effective population size is synchronous with the end of the glacial period observed in *C. glabrum* (Figure 4) and can be interpreted as postglacial expansion from glacial refugia. This suggests that these refugia harboured populations, which were smaller than the current populations, thus rejecting the hypothesis of major lowland refugia (Holderegger & Thiel‐Egenter, 2009) in the vicinity of the Pyrenees (e.g. Charrier et al., 2014; Schönswetter et al., 2004). The lack of an evident postglacial population growth in *S. borderei* can be explained by a strong postglacial migration lag, since most populations occur in previously non‐glaciated areas. In contrast, in *S. pyrenaica* major glacial refugia outside the ice sheet need to be invoked (Figure 2). However, we emphasize that uncertainty concerning mutation rate and generation time, as well as the moderate number of SNPs may result in inaccurate time estimates (Figure 4; Figure S6, Liu & Fu, 2015).

Even if compromised with uncertainties regarding the exact timing, the different pace of postglacial colonization and extent of postglacial range filling shaped the spatial genetic architecture of the studied species. The strong structure observed in *S. borderei* (Figures 2 and 3) and the strong differentiation between geographically close eastern populations (Figure S4) reveal long‐term persistence of populations in refugia in the eastern and western Pre‐Pyrenees. There, we observed the oldest divergence and the lowest levels of gene flow among the studied species, thus suggesting an enhanced role of genetic drift (Table S4, Sukumaran & Knowles, 2017). Similarly, *C. glabrum* showed a strong structure within the western Pyrenees (Figures 2 and 3). This supports the existence of multiple refugia in the western Pyrenees for *C. glabrum*, and in the eastern Pyrenees for *S. borderei*, as previously evidenced for several European mountain ranges including the Pyrenees (Bidegaray‐Batista et al., 2016; Kutnjak et al., 2014; Schönswetter et al., 2002). In contrast, the spatial genetic structure is weak in *S. pyrenaica*, where two groups were only observed in the STRUCTURE results (Figure 2), but not in the other analyses. Specifically, the neighbour‐net is star‐shaped with low degree of interpopulation differentiation as expected for expanding populations (Figure 2, Rogers & Harpending, 1992, Slatkin & Hudson, 1991). This pattern is likely further enhanced by a weaker effect of drift due to the younger divergence between the two groups and bigger effective population sizes as compared to *C. glabrum* and *S. borderei* (Table S4). A likely consequence of rapid expansion—and thus a high degree of range filling—is the formation of a hybrid zone in the central Pyrenees, observed as admixture cline (Figure 2) and evidenced by the highest between‐group migration rates across the study species (Table S4). Secondary contacts have often been observed in populations postglacially expanding from several refugia (Hewitt, 2001; Mayol et al., 2015; Schneeweiss et al., 2017; Winkler et al., 2012). Alternatively, the pattern of *S. pyrenaica* could be the result of isolation by distance (Wright, 1943), which we consider less likely as it would imply a very quick postglacial expansion from a single refugium, in contradiction with the large suitable area estimated under LGM conditions (Figure 2).

Habitat specialization strongly shapes postglacial colonization in the study species, partly overruling their dispersal abilities. This is reflected in the lesser extent of range filling and stronger genetic structure of the microsite specialists *C. glabrum* and *S. borderei*, as compared to *S. pyrenaica*, a species occupying a broader range of habitats. These results add to the suggestion of Dullinger et al. (2012) that other factors alongside dispersal ability play a major role in the postglacial expansion of alpine plants. Indeed, our results concur with the findings of a meta‐analysis of seed addition experiments (Clark et al., 2007), which concludes that in most cases establishment limitation prevails over seed limitation. Specifically, while plants might be able to disperse to new, climatically and geologically suitable habitats, a high degree of habitat specialization might challenge colonization success. In our case, although all studied species occur on calcicolous bedrock, *C. glabrum* grows exclusively in stable scree slopes with sparse vegetation cover and deep, nutrient‐rich soils, and *S. borderei* is found in crevices of vertical to overhanging cliffs (Talavera, 1990, 2014; P.C., own field observations), while *S. pyrenaica* can be found in a much wider range of habitats (Figure 5; Blanco, 2005). Thus, we suggest that the production of fruits adapted to wind dispersal in *C. glabrum* is obviously not sufficient to enable rapid colonization of its potential range, rendering this species a similarly poor colonizer as *S. borderei*, which is lacking apparent adaptations for long‐distance dispersal.

Our results, together with those of Clark et al. (2007), may explain the small effect of dispersal found in previous studies (e.g. Engler et al., 2009). However, we acknowledge some limitations of our migration‐based interpretation, since it is well known that other demographic parameters may have a strong influence on genetic drift and genetic structure (e.g. Dawson, 2014; Marko & Hart, 2011). For instance, the age of the study species is not known and the effective population sizes have been estimated with a high degree of uncertainty (Figure 4; Table S4). However, alongside the degree of range filling, the genetic structure and phylogenetic patterns, and the demographic models presented above, the roughly similar divergence times between genetic groups provide support for our conclusions.

Whereas we did not detect strong effects of dispersal ability on postglacial expansion on the range‐wide scale, it clearly drives small‐scale differences in spatial genetic patterns of *C. glabrum* and *S. borderei*. Specifically, the poor disperser *S. borderei* shows a higher level of local spatial genetic structure, a higher number of private alleles and a lower degree of admixture between groups as compared to *C. glabrum* (Figures 2 and 3; Figures S2 and S4). Dispersal hence appears important for the genetic make‐up of species within their current ranges, even if its impact on postglacial range expansion was limited. Overall, we show that species traits other than bedrock preference play an important role in determining the success of high‐elevation species tracking postglacial warming, challenging attempts of generalization (Alvarez et al., 2009). Among the studied traits, habitat specialization has more strongly shaped the postglacial colonization history of the study species than dispersal abilities. Thus, species occupying a broader range of habitats rapidly expanded and exchanged genes across populations after the end of the last glaciation, while populations of microsite specialists remained more isolated and in closer vicinity to their glacial refugia.

## CONFLICT OF INTEREST

None declared.

## BIOSKETCH

**Pau Carnicero** is an Assistant Professor at the Department of Botany, University of Innsbruck. He is particularly interested in the interaction between microevolution and environmental changes acting on mountain plants.

**Author contributions:** Pau Carnicero, Peter Schönswetter and Stefan Dullinger designed the study. Pau Carnicero conducted the genomic analyses. Dietmar Moser, Johannes Wessely and Stefan Dullinger performed the SDMs. Xavier Font prepared the vegetation database. Pau Carnicero and Peter Schönswetter wrote the first version of the manuscript. All authors commented on the manuscript.

## Supporting information


Figure S1
Figure S2
Figure S3
Figure S4
Figure S5
Figure S6
Figure S6

Table S1

Table S2

Table S3

Table S4

Appendix S5

## Data Availability

RADseq data are publicly available in the Sequence Read Archive (https://www.ncbi.nlm.nih.gov/sra), Bioproject PRJNA82209.
