# Supplementary material for: Postglacial range expansion of high‐elevation plants is restricted by dispersal ability and habitat specialization
Source: J Biogeogr. 2022 May 19;49(10):1739–52. doi: 10.1111/jbi.14390 (PMC9541807; doi:10.1111/jbi.14390)

**Postglacial range expansion of high-elevation plants is restricted by dispersal ability and habitat specialization**

Pau Carnicero, Johannes Wessely, Dietmar Moser, Xavier Font, Stefan Dullinger, Peter Schönswetter

**SUPPLEMENTARY FIGURES**

**Figure S1.** RAxML phylogenetic tree of *Cirsium glabrum* rooted with the outgroup. Bootstrap values above 50% are indicated above the branches. Numbers at the tips indicate the population numbers and the colored lines correspond to the most probable genetic group in STRUCTURE analyses at K = 2.

**
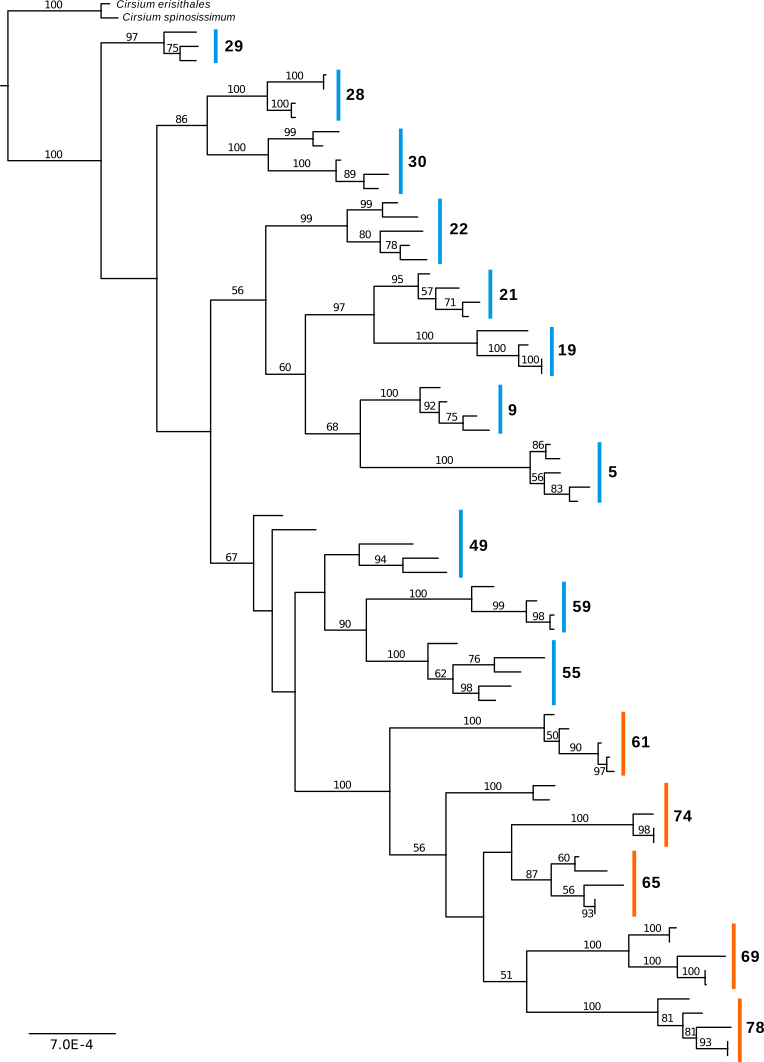
**

**Figure S2.** STRUCTURE admixture plots at K = 2 and K = 3 for the studied species. AT K = 2, blue corresponds to the western group and orange to the eastern group. Numbers below the plots indicate the population numbers.**
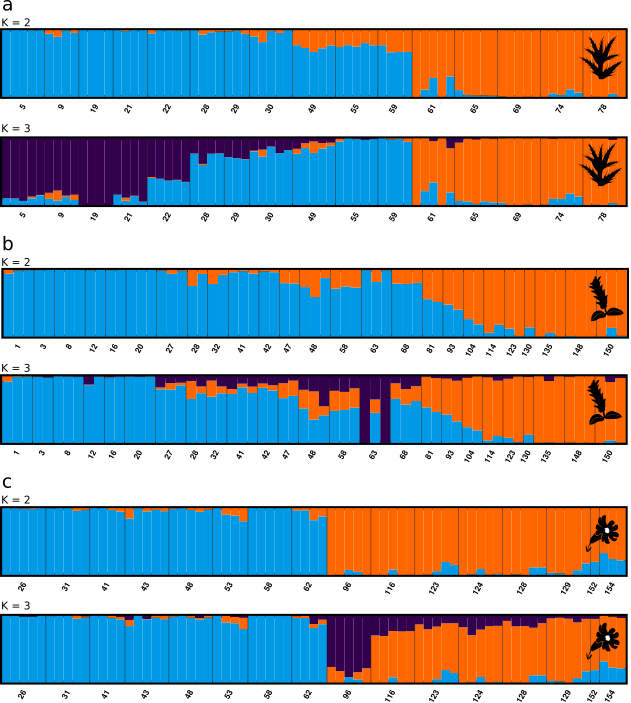
**

**Figure S3.** RAxML phylogenetic tree of *Silene borderei* rooted with the outgroup. Bootstrap values above 50% are indicated above the branches. Numbers at the tips indicate the population numbers and the colored lines correspond to the most probable genetic group in STRUCTURE analyses at K = 2.

**
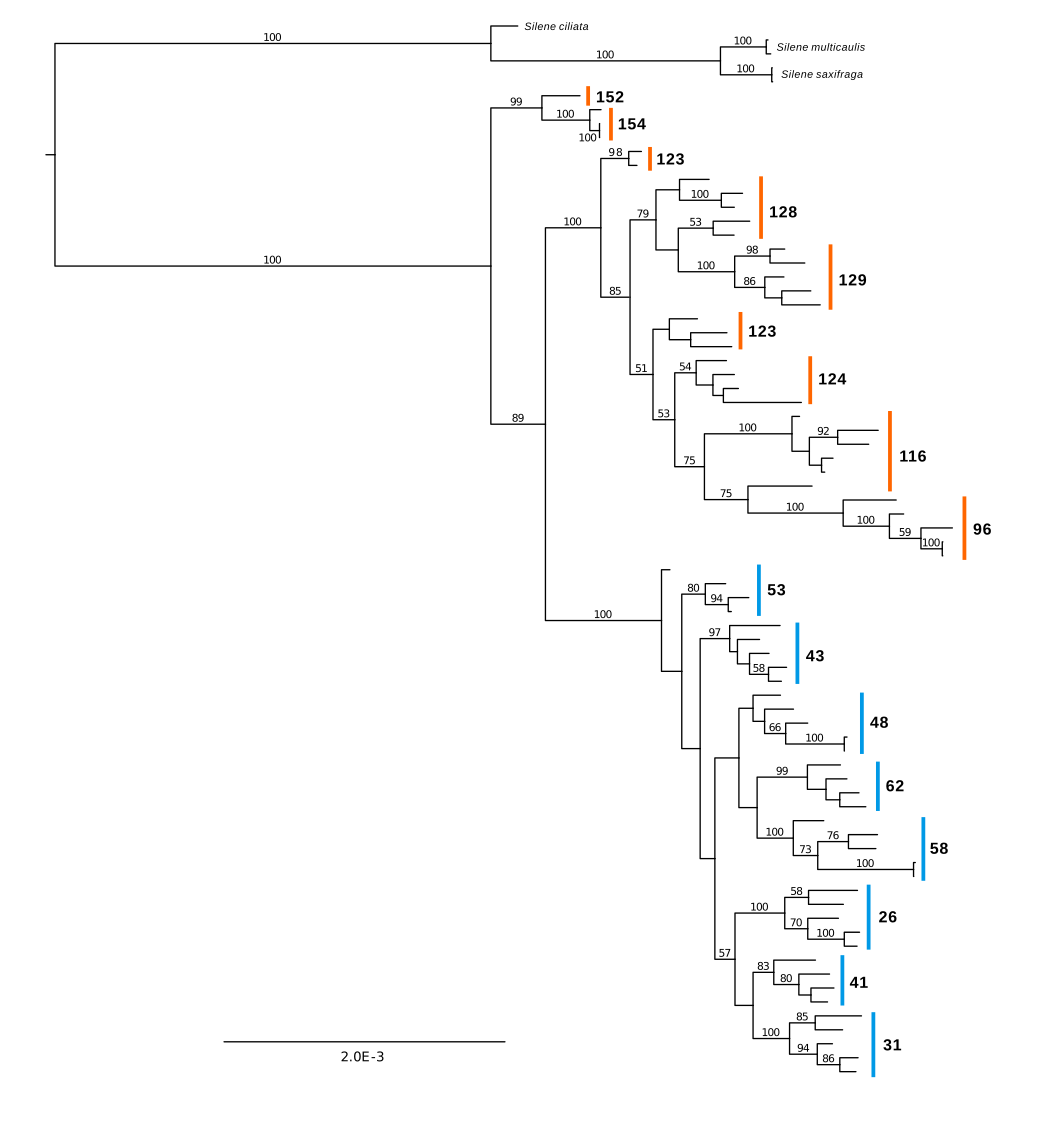
**

**Figure S4.** Genetic structure of the eastern group of *Silene borderei*. (a) collected populations (numbered), predicted suitable areas under current climatic conditions (red) and maximum extent of glaciers at the Last Glacial Maximum (white line). Maps generated with Lambert Azimuthal Equal Area projection, with datum ETRS89. (b) STRUCTURE admixture plots at K = 3 and K = 4. Numbers between the two plots indicate the population numbers. (c) FineRADstructure coancestry matrix. Blue indicates maximum levels of coancestry between two individuals, yellow the minimum (scale on the right). Numbers below the plot indicate the population numbers.

**
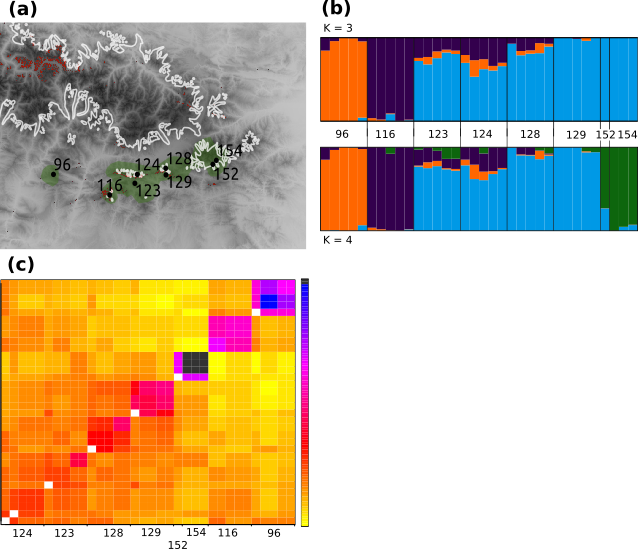
**

**Figure S5.** RAxML phylogenetic tree of *S. pyrenaica* rooted with the outgroup. Bootstrap values above 50% are indicated above the branches. Numbers at the tips indicate the population numbers and the colored lines correspond to the most probable genetic group in STRUCTURE analyses at K = 2. Populations painted in black had <75% assignation to a genetic group.

**
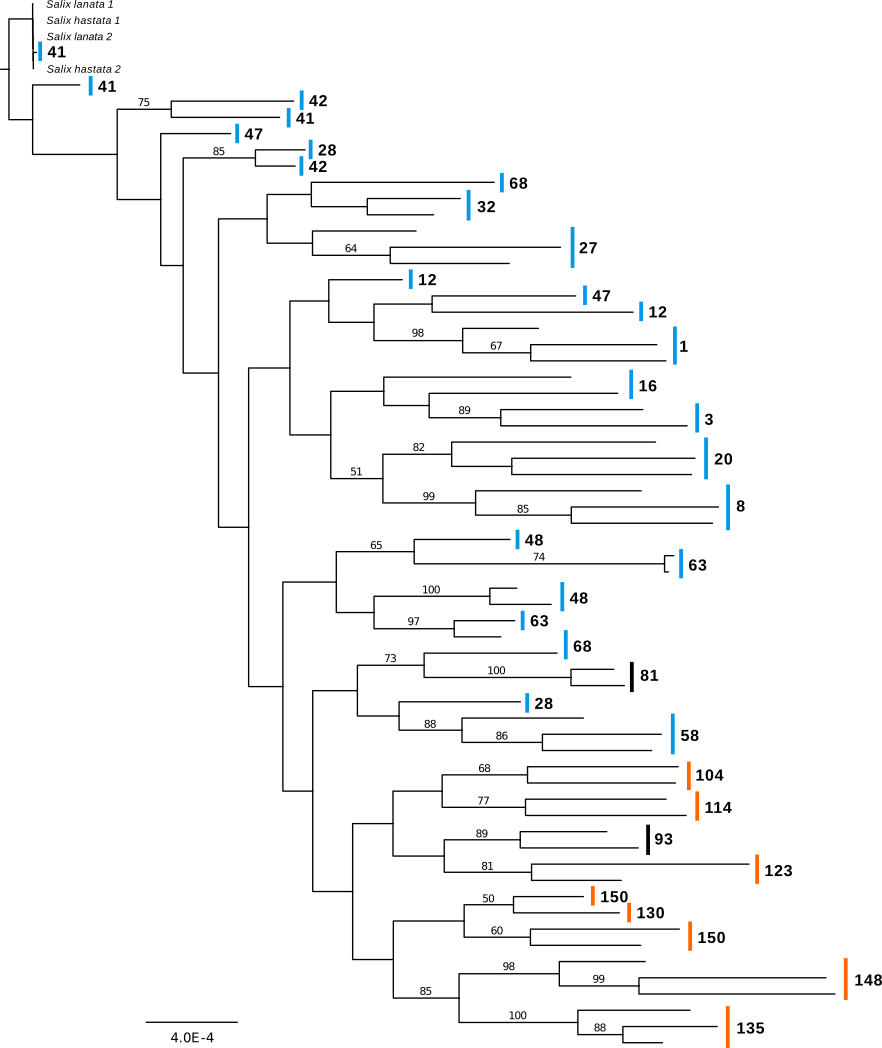
**

**Figure S6.** Changes in effective population size of the study species estimated with Stairway plots for different generation time and mutation rate values. gen, generation time in years; mu, mutation rate. Results for mutation rate 7 × 10^-9^ are shown in the main file. Colored lines indicate the genetic groups from STRUCTURE analyses at K = 2, black lines correspond to entire species. Dashed lines indicate the 95% confidence intervals of the median *Ne*. The dotted red vertical line indicates the Last Glacial Maximum. The left column shows the results for *Cirsium glabrum*, the middle *Salix pyrenaica*, and the right *Silene borderei*.

**
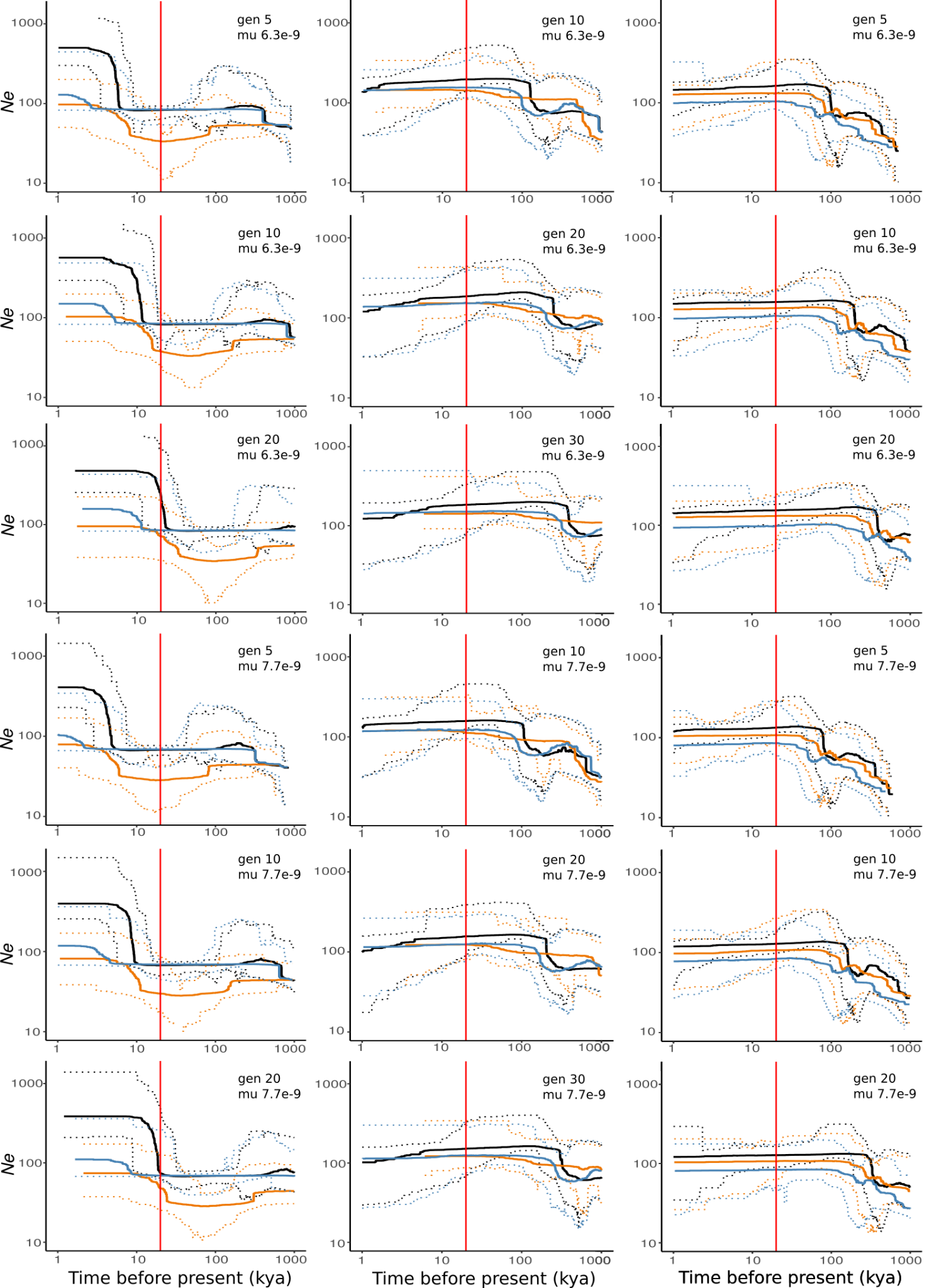
**

**Figure S7.** Demographic history of the study species. Best demographic models based on two-dimensional joint site frequency spectra (2D-SFS) between population sets defined by STRUCTURE analyses at K = 2. The two central columns show the observed and simulated 2D-SFS. Notice that due to the downprojection applied in the calculation of the site frequency spectra (see Material and Methods), the allele counts can be below 1. The third column shows the residuals, a standardized measure of how much the modelled 2D-SFS deviates from the observed 2D-SFS.


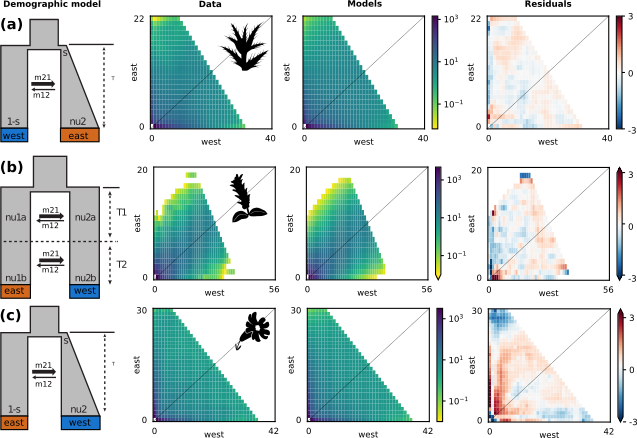

Supplement: Supplementary file 1 — Figure S1 Figure S2 Figure S3 Figure S4 Figure S5 Figure S6 Figure S6 [file JBI-49-1739-s004.docx]
