# Supplementary material for: Postglacial range expansion of high‐elevation plants is restricted by dispersal ability and habitat specialization
Source: J Biogeogr. 2022 May 19;49(10):1739–52. doi: 10.1111/jbi.14390 (PMC9541807; doi:10.1111/jbi.14390)
Supplement: Supplementary file 2 — Table S1 [file JBI-49-1739-s003.docx]

**Postglacial range expansion of high-elevation plants is restricted by dispersal ability and habitat specialization**

Pau Carnicero, Johannes Wessely, Dietmar Moser, Xavier Font, Stefan Dullinger, Peter Schönswetter

**SUPPLEMENTARY TABLE S1**

**Table S1.** Studied material. For each population, the code used in the manuscript and figures (ID) and collection data are indicated. Additionally the relative genome size ratio to *Bellis perennis*, the retrieved genetic groups from STRUCTURE analyses at K = 2, the allele diversity (ℼ) and the number of private alleles are shown. Short Read Archive (SRA) accession numbers for the RADseq data are given (SRA RAD; BioProject PRJNA82209; only last three digits of accession numbers starting with SAMN27176).

| Species | ID | Locality | Latitude | Longitude | Voucher | RGS to Bellis | genetic group | π | Private alleles | SRA RAD |
| --- | --- | --- | --- | --- | --- | --- | --- | --- | --- | --- |
| *Cirsium glabrum* | 5 | Spain, Aragón, La Jacetania, Zuriza, Llano Tatxeras | -0.794 | 42.854 | P. Carnicero 1634 (BCN164796) | 0.842 | W | 0.193 | 4 | 245-250 |
|  | 9 | Spain, Aragón, La Jacetania, Borau, Magdalena | -0.568 | 42.729 | P. Carnicero 1646 (BCN164795) | 0.819 | W | 0.228 | 0 | 251-257 |
|  | 19 | Spain, Aragón, Alto Gállego, Formigal | -0.372 | 42.776 | P. Carnicero 1868 (BCN164783) | 0.798 | W | 0.139 | 4 | 324-327 |
|  | 21 | Spain, Aragón, Alto Gállego, Hoz de Jaca, Pico Mandilar | -0.277 | 42.696 | P. Carnicero 1866 (BCN164784) | 0.810 | W | 0.207 | 0 | 319-323 |
|  | 22 | Spain, Aragón, Alto Gállego, Yésero, Puerto de Otal | -0.207 | 42.602 | P. Carnicero 1864 (BCN164785) | 0.785 | W | 0.220 | 0 | 314-318 |
|  | 28 | France, Occitanie, Gavarnie, Plateau de Pailla | 0.010 | 42.715 | P. Carnicero 1663 (BCN164797) | 0.841 | W | 0.224 | 0 | 258-262 |
|  | 29 | Spain, Aragón, Sobrarbe, Puértolas, Plana Canal | 0.105 | 42.591 | P. Carnicero 1862 (BCN164786) | 0.829 | W | 0.226 | 0 | 309-313 |
|  | 30 | Spain, Aragón, Sobrarbe, Valle de Pineta, Espierba | 0.122 | 42.675 | P. Carnicero 1697 (BCN164794) | 0.851 | W | 0.223 | 2 | 268-273 |
|  | 49 | Spain, Aragón, Sobrarbe, Saravillo, Ibón de Plan | 0.335 | 42.546 | P. Carnicero 1692 (BCN164798) | 0.823 | W | 0.270 | 2 | 263-267 |
|  | 55 | Spain, Aragón, Ribagorza, Barbaruens, Cotiella | 0.356 | 42.516 | P. Carnicero 1820 (BCN164789) | 0.820 | W |  |  | 294-298 |
|  | 59 | Spain, Aragón, Ribagorza, Sahún, Collado de Sahún - Val de Barbaricia | 0.406 | 42.579 | P. Carnicero 1850 (BCN164787) | 0.807 | W | 0.183 | 1 | 304-308 |
|  | 61 | Spain, Aragón, Ribagorza, Serrate, Turbón | 0.497 | 42.408 | P. Carnicero 1813 (BCN164791) | - | E | 0.178 | 3 | 284-288 |
|  | 65 | Spain, Aragón, Ribagorza, Cerler, coll de Basibé | 0.589 | 42.555 | P. Carnicero 1707 (BCN164793) | - | E | 0.217 | 0 | 274-278 |
|  | 69 | Spain, Aragón, Ribagorza, Benasque, Plan de l'Espital | 0.613 | 42.683 | P. Carnicero 1815 (BCN164790) | 0.829 | E | 0.185 | 4 | 289-293 |
|  | 74 | Spain, Aragón, Ribagorza, Aneto, Llauset | 0.692 | 42.585 | P. Carnicero 1810 (BCN164792) | 0.859 | E | 0.206 | 0 | 279-283 |
|  | 78 | Spain, Catalunya, Alta Ribagorça, Irgo, les Collades | 0.789 | 42.446 | P. Carnicero 1846 (BCN164788) | 0.802 | E | 0.153 | 9 | 299-303 |
| *Salix pyrenaica* | 1 | Spain, Navarra, Ochagavia, Monte Ori | -1.006 | 42.989 | P. Carnicero 1629 (BCN164782) | 0.277 | W | 0.213 | 1 | 410-412 |
|  | 3 | Spain, Aragón, La Jacetania, Isaba, Monte Lakora | -0.836 | 42.954 | P. Carnicero 1636 (BCN164781) | 0.277 | W | 0.224 | 0 | 413-415 |
|  | 8 | France, Occitanie, Forges d'Abel, Cabane Lapachouaou | -0.580 | 42.831 | P. Carnicero 1870 (BCN164754) | 0.280 | W | 0.216 | 0 | 484-486 |
|  | 12 | France, Occitanie, Laruns, Pic de Sesques | -0.498 | 42.929 | P. Carnicero 1657 (BCN164779) | 0.283 | W | 0.229 | 0 | 419-422 |
|  | 16 | Spain, Aragón, La Jacetania, Canfranc, Ip | -0.442 | 42.724 | P. Carnicero 1643 (BCN164780) | 0.290 | W | 0.230 | 0 | 416-418 |
|  | 20 | France, Occitanie, Gourette, Turon de Ger | -0.364 | 42.956 | P. Carnicero 1662 (BCN164778) | 0.273 | W | 0.228 | 0 | 423-425 |
|  | 27 | Spain, Aragón, Sobrarbe, Torla, Ordesa | -0.054 | 42.640 | P. Carnicero 1694 (BCN164775) | 0.276 | W | 0.232 | 0 | 433-435 |
|  | 28 | France, Occitanie, Gavarnie, Plateau de Pailla | 0.010 | 42.715 | P. Carnicero 1664 (BCN164777) | 0.280 | W | 0.236 | 0 | 426-428 |
|  | 32 | Spain, Aragón, Sobrarbe, Valle de Pineta, Espierba | 0.125 | 42.673 | P. Carnicero 1699 (BCN164773) | 0.273 | W | 0.231 | 0 | 439-441 |
|  | 41 | Spain, Aragón, Sobrarbe, Valle de Pineta, Portiello de Tella | 0.186 | 42.615 | P. Carnicero 1860 (BCN164756) | 0.287 | W | 0.223 | 0 | 481-183 |
|  | 42 | Spain, Aragón, Sobrarbe, Ceresa, Peña Montañesa | 0.203 | 42.489 | P. Carnicero 1696 (BCN164774) | 0.270 | W | 0.230 | 0 | 436-438 |
|  | 47 | France, Occitanie, Rioumajou, Tramezaigues, Lacs de Consaterre | 0.334 | 42.765 | P. Carnicero 1685 (BCN164776) | 0.273 | W | 0.229 | 0 | 429-432 |
|  | 48 | Spain, Aragón, Ribagorza, Barbaruens, Cotiella | 0.335 | 42.519 | P. Carnicero 1817 (BCN164759) | 0.270 | W | 0.226 | 0 | 469-471 |
|  | 58 | Spain, Aragón, Ribagorza, Sahún, Collado de Sahún | 0.402 | 42.569 | P. Carnicero (*no voucher*) | 0.267 | W | 0.221 | 0 | 476-480 |
|  | 63 | Spain, Aragón, Ribagorza, Serrate, Turbón | 0.509 | 42.413 | P. Carnicero 1811 (BCN164762) | 0.267 | W | 0.217 | 0 | 463-465 |
|  | 68 | Spain, Aragón, Ribagorza, Benasque, Plan de l'Espital | 0.612 | 42.684 | P. Carnicero 1814 (BCN164760) | 0.267 | W | 0.224 | 0 | 466-468 |
|  | 81 | Spain, Catalunya, Vall d'Aran, Valarties, Camin de Rius | 0.867 | 42.650 | P. Carnicero 1731 (BCN164770) | 0.283 | admixed | 0.205 | 0 | 442-444 |
|  | 93 | Spain, Catalunya, Pallars Sobirà, Espot, above Prat del Pierró | 1.047 | 42.579 | P. Carnicero 1845 (BCN164757) | 0.273 | admixed | 0.219 | 0 | 475-477 |
|  | 104 | France, Occitanie, Vicdessos, pr. Col d'Agnes | 1.368 | 42.797 | P. Carnicero 1759 (BCN164769) | 0.280 | E | 0.219 | 0 | 445-447 |
|  | 114 | Andorra, Pal, Alt de la Capa | 1.447 | 42.561 | P. Carnicero 1828 (BCN164758) | 0.273 | E | 0.216 | 0 | 472-474 |
|  | 123 | Spain, Catalunya, Berguedà, Saldes, Pedraforca | 1.699 | 42.244 | P. Carnicero 1800 (BCN164766) | 0.317 | E | 0.235 | 0 | 454-456 |
|  | 130 | Spain, Catalunya, Berguedà, Bagà, Roc dels Canells | 1.908 | 42.300 | P. Carnicero 1797 (BCN164767) | 0.265 | E | 0.246 | 0 | 451-453 |
|  | 135 | France, Occitanie, La Fajolle, Col de Pailhères | 1.996 | 42.741 | P. Carnicero 1801 (BCN164765) | 0.270 | E | 0.211 | 0 | 457-459 |
|  | 148 | France, Occitanie, Oleta, above Pla de la Velleta | 2.213 | 42.621 | P. Carnicero 1804 (BCN164764) | 0.267 | E | 0.224 | 0 | 460-462 |
|  | 150 | Spain, Catalunya, Ripollès, Queralbs, Gorges del Freser | 2.216 | 42.382 | P. Carnicero 1792 (BCN164768) | 0.263 | E | 0.228 | 0 | 448-450 |
| *Silene borderei* | 26 | Spain, Aragón, Sobrarbe, Torla, Valle de Ordesa | -0.055 | 42.639 | P. Carnicero (*no voucher*) | 1.435 | W | 0.183 | 8 | 395-399 |
|  | 31 | Spain, Aragón, Sobrarbe, Valle de Pineta, Espierba | 0.124 | 42.674 | P. Carnicero 1698 (BCN164811) | 1.435 | W | 0.195 | 0 | 334-338 |
|  | 41 | Spain, Aragón, Sobrarbe, Valle de Pineta, Portiello de Tella | 0.186 | 42.615 | P. Carnicero 1859 (BCN164800) | 1.440 | W | 0.196 | 0 | 390-394 |
|  | 43 | Spain, Aragón, Sobrarbe, Ceresa, Peña Montañesa | 0.206 | 42.491 | P. Carnicero 1695 (BCN164812) | 1.435 | W | 0.214 | 0 | 329-333 |
|  | 48 | Spain, Aragón, Ribagorza, Barbaruens, Cotiella | 0.335 | 42.519 | P. Carnicero 1818 (BCN164804) | 1.440 | W | 0.217 | 3 | 370-374 |
|  | 53 | Spain, Aragón, Sobrarbe, Saravillo | 0.342 | 42.543 | P. Carnicero (*no voucher*) | 1.435 | W | 0.184 | 7 | 339-343 |
|  | 58 | Spain, Aragón, Ribagorza, Sahún, Collado de Sahún | 0.402 | 42.569 | P. Carnicero 1851 (BCN164801) | 1.445 | W |  |  | 385-389 |
|  | 62 | Spain, Aragón, Ribagorza, Serrate, Turbón | 0.505 | 42.417 | P. Carnicero 1812 (BCN164805) | 1.425 | W | 0.209 | 1 | 365-369 |
|  | 96 | Spain, Catalunya, Pallars Jussà, Serra de Boumort, Hortoneda | 1.132 | 42.237 | P. Carnicero 1832 (BCN164802) | 1.435 | E | 0.207 | 14 | 380-384 |
|  | 116 | Spain, Catalunya, Solsonès, Port del Compte, Coll de Tancalaporta | 1.533 | 42.175 | P. Carnicero 1821 (BCN164803) | 1.420 | E | 0.204 | 7 | 375-379 |
|  | 123 | Spain, Catalunya, Berguedà, Saldes, Pedraforca | 1.699 | 42.244 | P. Carnicero 1799 (BCN164807) | 1.430 | E | 0.259 | 5 | 355-359 |
|  | 124 | Spain, Catalunya, La Cerdanya, Cadí, Montellà, Pas dels Gosolans | 1.710 | 42.287 | P. Carnicero 1805 (BCN164806) | 1.425 | E |  |  | 360-364 |
|  | 128 | Spain, Catalunya, La Cerdanya, Masella, Tossa d'Alp | 1.902 | 42.331 | P. Carnicero 1773 (BCN164810) | 1.440 | E | 0.253 | 24 | 344-348 |
|  | 129 | Spain, Catalunya, Berguedà, Bagà, Roc dels Canells | 1.908 | 42.301 | P. Carnicero 1795 (BCN164808) | 1.420 | E |  |  | 350-354 |
|  | 152 | Spain, Catalunya, Ripollès, Queralbs, Balandrau | 2.225 | 42.375 | P. Carnicero 1790 (BCN164809) | - | E | 0.228 | 26 | 349 |
|  | 154 | Spain, Catalunya, Ripollès, Setcases, Vall de l'Orri | 2.247 | 42.396 | M. Guardiola (BCN164799) | - | E |  |  | 400-402 |
| Outgroups |  |  |  |  |  |  |  |  |  |  |
| *Cirsium erisithales* |  | Austria, Kärnten, Lienzer Dolomiten, Anetwände (cultivated in the Botanical Garden of the University of Innsbruck (18756)) | 12.903 | 46.771 | *no voucher* |  |  |  |  | 244 |
| *Cirsium spinosissimum* |  | Austria, Tirol, Tuxer Alpen, Schmirntal (cultivated in the Botanical Garden of the University of Innsbruck (21480)) | 11.630 | 47.086 | *no voucher* |  |  |  |  | 328 |
| *Salix hastata 1* |  | Austria, Tirol, Ötztaler Alpen, Obergurgl | 11.048 | 46.768 | E. Hörandl, S. Hörandl, F. Hadacek 10246 |  |  |  |  | 404 |
| *Salix hastata 2* |  | Italy, Südtirol, Vingschau, Stilfserjoch | 10.509 | 46.451 | E. Hörandl, S. Hörandl, F. Hadacek 10259 |  |  |  |  | 405 |
| *Salix lanata 1* |  | Norway, Sogn og Fjordane, Aurland, Aurlandsdalen | 8.706 | 58.777 | E. Hörandl, S. Hörandl, F. Hadacek 10215 |  |  |  |  | 406 |
| *Salix lanata 2* |  | Norway, Hordaland, Hardangervidda, Skuleiyikstølen | 8.740 | 58.567 | E. Hörandl, S. Hörandl, F. Hadacek 10221 |  |  |  |  | 407 |
| *Silene ciliata* |  | France, Occitanie, Font Romeu, Carlit | 19.413 | 42.570 | P. Carnicero 1872 (BCN164814) |  |  |  |  | 403 |
| *Silene multicaulis* |  | Montenegro, Prokletije, Žijevo | 19.481 | 42.553 | P. Schönswetter, B. Frajman & Denis Kutnjak 13009 |  |  |  |  | 408-409 |
| *Silene saxifraga* |  | Italy, Cuneo, Alpi Maritimi, Tetti Violino | 7.427 | 44.245 | B. Frajman 14164 |  |  |  |  | 487, 488 |
