# Supplementary material for: Postglacial range expansion of high‐elevation plants is restricted by dispersal ability and habitat specialization
Source: J Biogeogr. 2022 May 19;49(10):1739–52. doi: 10.1111/jbi.14390 (PMC9541807; doi:10.1111/jbi.14390)
Supplement: Supplementary file 3 — Table S2 [file JBI-49-1739-s005.docx]

**Table S2.** Filters applied and resulting numbers of SNPs and individuals for the employed genomic analyses. For the filters applied, the program (Populations in Stacks or VCFtools) and the flag used in the analysis scripts are indicated. The True Skill Statistic scores (TSS) of the ensemble models from the species distribution models are also indicated. inds, number of individuals.

|  |  | *Cirsium glabrum* | *Silene borderei* | *Salix pyrenaica* |
| --- | --- | --- | --- | --- |
|  | Maximum missing data per individual | 50% | 40% | 50% |
| Neighbor-net | populations --min_maf | 3 inds | 3 inds | 3 inds |
|  | populations --max_obs_het | 65% | 65% | 65% |
|  | vcftools --minDP | 10 | 10 | 10 |
|  | vcftools --max-missing | 80 | 80 | 50 |
|  | SNPs | 3407 | 7558 | 72525 |
|  | loci | 2232 | 5068 | 29549 |
| RAxML | populations --min_maf | 3 inds | 3 inds | 3 inds |
|  | populations --max_obs_het | 65% | 65% | 65% |
|  | vcftools --minDP | 10 | 10 | 8 |
|  | vcftools --max-missing | 80 | 80 | 80 |
|  | SNPs | 3615 | 11598 | 17703 |
| STRUCTURE | populations --min_maf | 3 inds | 3 inds | 3 inds |
|  | populations --max_obs_het | 65% | 65% | 65% |
|  | populations -R | 0.8 | 0.8 | 0.8 |
|  | SNPs | 4096 | 6873 | 9602 |
| fineRADstructure | SNPs | 6293 | 16566 | 48366 |
| popgen stats /group | populations --min_maf | 3 inds | 3 inds | 3 inds |
|  | populations --max_obs_het | 65% | 65% | 65% |
|  | populations -r | 0.8 | 0.8 | 0.8 |
|  | populations -R | 0.8 | 0.8 | 0.8 |
|  | SNPs | 6293 | 12849 | 35463 |
| SFS Stairway plot | populations --max_obs_het | 65% | 65% | 65% |
|  | vcftools --minDP | 10 | 10 | 10 |
|  | --maxDP | 50 | 50 | 50 |
|  | --minGQ | 20 | 20 | 20 |
|  | vcftools --max-missing (stairway) | 0.7 | 0.7 | 0.7 |
|  | SNPs (all/east/west) | 6524 / 6937 / 7238 | 16886 / 15375 / 19332 | 24165 / 21406 / 25677 |
| SFS dadi | populations --max_obs_het | 65% | 65% | 65% |
|  | vcftools --minDP | 10 | 10 | 10 |
|  | --maxDP | 50 | 50 | 50 |
|  | --minGQ | 20 | 20 | 20 |
|  | vcftools --max-missing | 0.5 | 0.5 | 0.7 |
|  | SNPs | 9567 | 23001 | 26148 |
|  | alleles after downprojection (east/west) | 22/40 | 30/42 | 20/56 |
| SDMs | TSS ensemble model | 0.94 | 0.99 | 0.99 |
|  |  |  |  |  |
|  |  |  |  |  |
