# Supplementary material for: Postglacial range expansion of high‐elevation plants is restricted by dispersal ability and habitat specialization
Source: J Biogeogr. 2022 May 19;49(10):1739–52. doi: 10.1111/jbi.14390 (PMC9541807; doi:10.1111/jbi.14390)
Supplement: Supplementary file 5 — Table S4 [file JBI-49-1739-s002.docx]

**Table S4.** Inferred parameters of demographic models based on two-dimensional joint site frequency spectra between population sets defined by STRUCTURE analyses at K = 2. The selected models for each species are indicated in the text and in Figure S7. *N*, effective population size in number of individuals, indicating the initial values before divergence (*N_0_*), the values of each genetic group during the first epoch in case of *Salix pyrenaica*, or the initial value for the founded population for *Cirsium glabrum* and *Silene borderei* (*N_1_*), and the current values of each genetic group (E, eastern; W, western); *T0*, time to divergence; *T2*, time to most recent change in population size after divergence (only for *S. pyrenaica*); *m*, migration rate as proportion of individuals migrating from the source population (E-W, from eastern to western group; W-E, from western to eastern group).

|  | *Cirsium glabrum* | *Silene borderei* | *Salix pyrenaica* |
| --- | --- | --- | --- |
| *N_0_* | 4946 | 20075 | 17050 |
| *N_1E_* | 2463 | - | 15918 |
| *N_1W_* | - | 207 | 27594 |
| *N_E_* | 10880 | 19868 | 25308 |
| *N_W_* | 2483 | 18748 | 39477 |
| *T_0_* (Kya) | 530 | 681 | 419 |
| *T_2_* (Kya) | - | - | 251 |
| *m_E-W_* | 8.6 ✕ 10^-5^ | 3.6 ✕ 10^-5^ | 3.1 ✕ 10^-4^ |
| *m_W-E_* | 2.3 ✕ 10^-4^ | 2.3 ✕ 10^-5^ | 4.3 ✕ 10^-5^ |
