# Supplementary material for: Postglacial range expansion of high‐elevation plants is restricted by dispersal ability and habitat specialization
Source: J Biogeogr. 2022 May 19;49(10):1739–52. doi: 10.1111/jbi.14390 (PMC9541807; doi:10.1111/jbi.14390)
Supplement: Supplementary file 6 — Appendix S5 [file JBI-49-1739-s001.docx]

**Postglacial range expansion of high-elevation plants is restricted by dispersal ability and habitat specialization**

Pau Carnicero, Johannes Wessely, Dietmar Moser, Xavier Font, Stefan Dullinger, Peter Schönswetter

**SUPPLEMENTARY METHODS**

**Environmental variables: details about the downscaling procedure**

We applied a statistical downscaling procedure previously used in several studies on climate change effects (Zimmermann et al., 2009; Ramirez-Villegas & Jarvis, 2010; Tabor & Williams, 2010; Dullinger et al.*,* 2012). The “delta-method” uses deviations (i.e., deltas) between historic and current climatic conditions within predicted (i.e., hind-casted) time series, which are spatially interpolated from their (coarser) spatial resolution to those of current data series based on measurements using a cubic-spline algorithm and, finally, added to the latter. As historic data we used reconstructions of the paleoclimate based on the Community Climate System Model ver. 3 (CCSM3) with a spatial resolution of 2.5°. These data emerged from the TRaCE21ka experiment (Liu et al., 2009; Otto-Bliesner et al., 2014) and is accessible via PaleoView (Fordham et al., 2017). Current climatic conditions with a spatial resolution of 30″ (i.e. approximately 1 km within the study area) were retrieved from the CHELSA Climate database (Karger et al., 2017a, 2017b) available at <http://chelsa-climate.org/>. Due to the short temporal overlap between CHELSA data (representing averages of the years 1979-2013) and the paleoclimate (ending 1983) the delta-method was applied in a two-step approach including climate data having a temporal overlap of some decades with both data sets became necessary. We used the CRU TS4.01 data set (Harris et al., 2014) with a spatial resolution of 0.5° covering the period 1901-2016. As the first step we calculated arithmetic means of climate variables (i.e., monthly precipitation as well as minimum, maximum and average monthly temperature) of the paleoclimate data representing the LGM (i.e., 30 years centred around 21000 BP) and recent climatic conditions (i.e., 1979–2013). The latter is used as reference period to enable the link to the CRU data. Further, we defined the deltas of climate variables as differences between historic and current (minimum, maximum and average) temperature as well as the ratio (historic/current) in case of average precipitation. These deltas were computed between the LGM and the 1950-1980 reference period at 2.5° resolution, spatially interpolated to 0.5° to match the resolution of the CRU data using thin plate spline method and added to the CRU data. This procedure was repeated in a second step of the downscaling approach to further link the modified CRU data (0.5°) to the CHELSA climatologies (30″) by calculating deltas of climate variables of the modified CRU data between the LGM and the reference period of 1979-2013, interpolated these deltas to the resolution of the CHELSA climatologies, and adding them to the CHELSA data. We used these downscaled monthly projections of climate variables to calculate two bioclimatic variables representing climatic conditions during the growing season: mean temperature of the warmest quarter (bio10) and precipitation sum of the warmest quarter (bio18). These climatic variables were projected to a grid of 1x1 km cell size using the nearest neighbour method.

Dullinger, S., Gattringer, A., Thuiller, W., Moser, D., Zimmermann, N. E., Guisan, A., Willner, W., Plutzar, C., Leitner, M., Mang, T., Caccianiga, M., Dirnbock, T., Ertl, S., Fischer, A., Lenoir, J., Svenning, J. C., Psomas, A., Schmatz, D. R., Silc, U., Vittoz, P., & Hulber, K., (2012). Extinction debt of high-mountain plants under twenty-first-century climate change. Nature Climate Change 2, 619-622.

Fordham, D. A., Saltre, F., Haythorne, S., Wigley, T. M. L., Otto-Bliesner, B. L., Chan, K. C., Brook, & B. W., (2017). PaleoView: a tool for generating continuous climate projections spanning the last 21000 years at regional and global scales. Ecography 40, 1348-1358.

Harris, I., Jones, P. D., Osborn, T. J., & Lister, D. H., (2014). Updated high-resolution grids of monthly climatic observations - the CRU TS3.10 Dataset. International Journal of Climatology 34, 623-642.

Karger, D. N., Conrad, O., Böhner, J., Kawohl, T., Kreft, H., Soria-Auza, R. W., Zimmermann, N. E., Linder, H. P. & Kessler, M. (2017a) Climatologies at high resolution for the earth’s land surface areas. Scientific Data 4, 170122. <https://doi.org/10.1038/sdata.2017.122>

Karger, D. N., Conrad, O., Böhner, J., Kawohl, T., Kreft, H., Soria-Auza, R. W., Zimmermann, N. E., Linder, H. P. & Kessler, M. (2017b). Data from: Climatologies at high resolution for the earth’s land surface areas. Dryad Digit. Repos. https://doi.org/10.5061/dryad.kd1d4

Liu, Z., Otto-Bliesner, B. L., He, F., Brady, E. C., Tomas, R., Clark, P. U., Carlson, A. E., Lynch-Stieglitz, J., Curry, W., Brook, E., Erickson, D., Jacob, R., Kutzbach, J., & Cheng, J., (2009). Transient simulation of last deglaciation with a new mechanism for Bolling-Allerod warming. Science 325, 310-314.

Otto-Bliesner, B. L., Russell, J. M., Clark, P. U., Liu, Z. Y., Overpeck, J. T., Konecky, B., deMenocal, P., Nicholson, S. E., He, F., & Lu, Z. Y., (2014). Coherent changes of southeastern equatorial and northern African rainfall during the last deglaciation. Science 346, 1223-1227.

Ramirez-Villegas, J., & Jarvis, A., (2010. Downscaling global circulation model outputs: the delta method decision and policy analysis working paper No. 1. International Center for Tropical Agriculture, CIAT, Cali, Colombia.

Tabor, K., & Williams, J. W., (2010). Globally downscaled climate projections for assessing the conservation impacts of climate change. *Ecological Applications*, 20, 554-565.

Zimmermann, N. E., Yoccoz, N. G., Edwards, T. C., Meier, E. S., Thuiller, W., Guisan, A., Schmatz, D. R., Pearman, P. B., (2009). Climatic extremes improve predictions of spatial patterns of tree species. *Proceedings of the National Academy of Sciences of the United States of America*, 106(2), 19723–19728
